# Global patterns of syphilis, gonococcal infection, typhoid fever, paratyphoid fever, diphtheria, pertussis, tetanus, and leprosy from 1990 to 2021: findings from the Global Burden of Disease Study 2021

**DOI:** 10.1186/s40249-024-01231-2

**Published:** 2024-09-13

**Authors:** Weiye Chen, Yiming Chen, Zile Cheng, Yiwen Chen, Chao Lv, Lingchao Ma, Nan Zhou, Jing Qian, Chang Liu, Min Li, Xiaokui Guo, Yongzhang Zhu

**Affiliations:** 1 School of Global Health, Chinese Center for Tropical Diseases Research, Shanghai Jiao Tong University School of Medicine, Shanghai, China; 2 School of Public Health, Shanghai Jiao Tong University School of Medicine, Shanghai, China; 3 Key Laboratory of Parasite and Vector Biology, National Institute of Parasitic Diseases, Chinese Center for Disease Control and Prevention (Chinese Center for Tropical Diseases Research), Shanghai, 200025 China; 4 Department of Immunology and Microbiology, Shanghai Jiao Tong University School of Medicine, Shanghai, China

**Keywords:** Syphilis, Gonococcal infection, Typhoid and paratyphoid fever, Leprosy, Pertussis, Diphtheria, Tetanus, Global burden of disease, Disability-adjusted life-years, Socio-demographic index

## Abstract

**Background:**

Certain infectious diseases are caused by specific bacterial pathogens, including syphilis, gonorrhea, typhoid and paratyphoid fever, diphtheria, pertussis, tetanus, leprosy, and tuberculosis. These diseases significantly impact global health, contributing heavily to the disease burden. The study aims to thoroughly evaluate the global burden of syphilis, gonorrhea, typhoid and paratyphoid fever, diphtheria, pertussis, tetanus, and leprosy.

**Methods:**

Leveraging the Global Burden of Disease (GBD) study 2021, age-specific and Socio-demographic Index (SDI)-specific incidence, disability-adjusted life-years (DALYs), and death for eight specific bacterial infections across 204 countries and territories from 1990 to 2021 were analyzed. Percentage changes in age-standardized incidence rate (ASIR), DALY rate, and mortality rate (ASMR) were also examined, with a focus on disease distribution across different regions, age groups, genders, and SDI.

**Results:**

By 2021, among the eight diseases, gonococcal infection had the highest global ASIR [1096.58 per 100,000 population, 95% uncertainty interval (UI): 838.70, 1385.47 per 100,000 population], and syphilis had the highest global age-standardized DALY rate (107.13 per 100,000 population, 95% UI: 41.77, 212.12 per 100,000 population). Except for syphilis and gonococcal infection, the age-standardized DALY rate of the remaining diseases decreased by at least 55% compared to 1990, with tetanus showing the largest decrease by at least 90%. Globally, significant declines in the ASIR, age-standardized DALY rate, and ASMR for these eight bacterial infections have been observed in association with increases in the SDI. Regions with lower SDI, such as sub-Saharan Africa, experienced a relatively higher burden of these eight bacterial infections.

**Conclusions:**

Although there has been an overall decline in these eight diseases, they continue to pose significant public health challenges, particularly in low SDI regions. To further reduce this burden in these areas, targeted intervention strategies are essential, including multi-sectoral collaboration, policy support, improved WASH management, and enhanced research efforts.

**Graphical Abstract:**

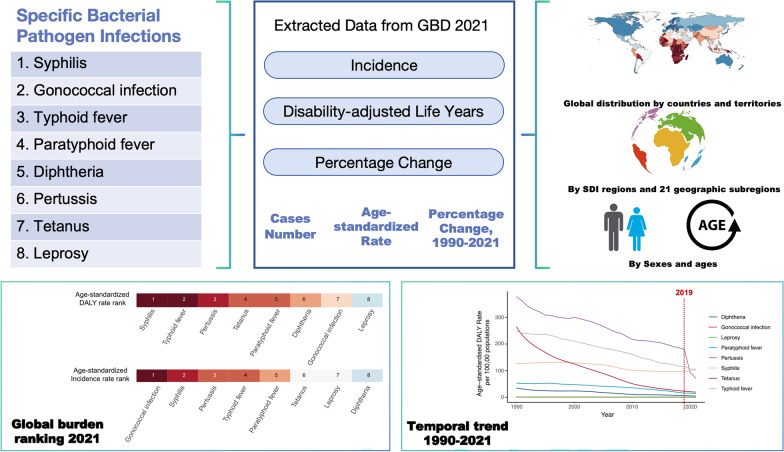

**Supplementary Information:**

The online version contains supplementary material available at 10.1186/s40249-024-01231-2.

## Background

Bacterial pathogens serve as significant contributors to the occurrence of infectious diseases. Some diseases, such as meningitis, can be caused by multiple pathogens including *Streptococcus pneumoniae, Neisseria meningitidis*, and even viruses [1, 2]. However, it is noteworthy that certain infectious diseases are attributed to specific bacterial pathogens. These include syphilis, gonorrhea, typhoid and paratyphoid fevers, diphtheria, pertussis, tetanus, leprosy, and tuberculosis (TB).

Numerous studies have convincingly demonstrated that these nine specific diseases exert a profound impact on the global burden of disease [3–7]. Specifically, sexually transmitted infections (STIs) such as syphilis and gonorrhea, caused by *Treponema pallidum* and *Neisseria gonorrhoeae* respectively, can lead to infertility, genital damage, and an increased risk of human immunodeficiency virus (HIV) infection [8, 9]. Typhoid and paratyphoid fever, diseases of the intestinal system, are caused by *Salmonella enterica* subspecies serovars Typhi and Paratyphi [10]. The global burden of typhoid fever is estimated to be about 21.7 million cases and 217,000 fatalities annually [11]. Leprosy, a neglected tropical disease, is caused by the *Mycobacterium leprae* [12]. Fortunately, the global incidence of leprosy has decreased by 27.86% from 1990 to 2019 following the implementation of multidrug therapy by the World Health Organization (WHO) [5, 13]. Diphtheria, pertussis, and tetanus are caused by *Corynebacterium diphtheriae, Bordetella pertussis*, and *Clostridium tetani*, respectively. Despite the effectiveness of the diphtheria-tetanus-pertussis (DTP) vaccine in preventing and controlling these diseases, complete elimination remains unattainable at present [14]. Tuberculosis, caused by *Mycobacterium tuberculosis*, stands as a formidable global health issue, responsible for a considerable number of deaths, ranking second only to coronavirus disease 2019 (COVID-19) in terms of mortality rates [15]. The WHO has implemented corresponding measures to combat these nine diseases, including strategies to end STIs, TB, and leprosy, as well as efforts to strengthen vaccine coverage, including the DTP, typhoid, and paratyphoid vaccines [16–21].

The Global Burden of Disease (GBD) 2021 database is designed to assess the global burden of 371 diseases in 204 countries and territories from 1990 to 2021 [22]. According to the GBD study 2021, estimates of the disease burden for TB in 2021 have been thoroughly investigated [7]. It is also necessary to estimate the disease burden of eight other specific bacterial infections by country, region, age, sex, and SDI, particularly to provide information on the prevalence during the COVID-19 pandemic (after 2019). In this study, the results of GBD 2021 were utilized to estimate the burden of these eight specific bacterial pathogens, analyzing epidemiological data to determine transmission trends at global and regional levels.

## Methods

### Data source

The data for this research were sourced from the Institute for Health Metrics and Evaluation (IHME) through their official website at https://ghdx.healthdata.org/gbd-results/. The specific search criteria used in the “Search” interface were as described below: GBD Estimate (cases of death or injury, risk factor), Measure (incidence, deaths, DALY, prevalence), Metric (number, percent, rate), Cause (syphilis, gonococcal infection, typhoid fever, paratyphoid fever, diphtheria, pertussis, tetanus, leprosy). Location (global, all countries and regions, different Socio-demographic Index [SDI] regions, 204 countries and territories), Age (all ages, age-standardized, < 5 years, 5–9 years, 10–14 years, 15–19 years, 20–24 years, 25–29 years, 30–34 years, 35–39 years, 40–44 years, 45–49 years, 50–54 years, 55–59 years, 60–64 years, 65–69 years, 70–74 years, 75–79 years, 80–84 years, 85–89 years, 90–94 years, > 95 years old), Sex (both, male, female), Year (1990–2021).

### Case definition

The study focused on eight infectious diseases that were clearly diagnosed as specific bacterial infections. They were syphilis (*Treponema pallidum*), gonococcal infection (*Neisseria gonorrhoea*), typhoid fever (*Salmonella enterica typhi*), paratyphoid fever (*Salmonella enterica paratyphi*), diphtheria (*Corynebacterium diphtheriae*), pertussis (*Bordetella pertussis*), tetanus (*Clostridium tetani*), and leprosy (*Mycobacterium leprae*). For syphilis, the International Classification of Diseases (ICD)-10 codes are A50–A53.9, I98.0, K67.2, M73.1–M73.8, and ICD-9 codes are 090–097.9. For gonococcal infection, ICD-10 codes are A54–A54.9, K67.1, M73.0, N74.3, and ICD-9 codes are 098–098.9. For typhoid fever, ICD-10 codes are A01.0–A01.09, and ICD-9 codes are 002.0. For paratyphoid fever, ICD-10 codes are A01.1–A01.4, and ICD-9 codes are 002.1–002.9. For diphtheria, ICD-10 codes are A36–36.9, and ICD-9 codes are 032–032.9, V02.4, V03.5, V74.3. For pertussis, ICD-10 codes are A37–A37.91, and ICD-9 codes are 033–033.9, V03.6. For tetanus, ICD-10 codes are A33–A35.0, ICD-9 codes are 037–037.9, 771.3, V03.7. For leprosy, ICD-10 codes are A30–A30.9, B92 and ICD-9 codes are 030–030.9, V74.2.

### Statistical analysis

Age-standardized rate (ASR) per 100,000 population were extracted from the GBD 2021 database, including age-standardized incidence rate (ASIR), age-standardized DALY rate, and age-standardized mortality rate (ASMR). The ASR is calculated based on the age structure of standard populations using the following formula:

$$ASR = \frac{{\sum\nolimits_{i = 1}^{N} {a_{i} w_{i} } }}{{\sum\nolimits_{i = 1}^{N} {wi} }}$$

where $a_{i}$ is the age-specific rate in the $i$th age group, $w_{{\text{i}}}$ is the number of persons (or the weight) in the corresponding $i$th age subgroup of the selected reference standard population, and $N$ is the number of age groups. The 95% uncertainty intervals (UIs) were defined as the 2.5th and 97.5th values of the ordered 1000 draws.

The GBD database utilizes UIs rather than precise statistical values. Therefore, in this study, statistical significance cannot be directly calculated when comparing two numerical values, like numbers and rates; only UIs are provided. If the UIs overlap, it suggests no significant difference (*P* > 0.05). On the other hand, if the UIs do not overlap, it indicates a significant difference (*P* < 0.05).

Smoothing spline models were used to evaluate the relationship between the burdens of the eight diseases and SDI across the 21 geographic regions and 204 countries and territories. The expected values were determined through calculations that consider the SDI and disease rates across all locations. Smooth splines were applied using the ggplot2 with geom_smooth by method “loess” [23].

All statistical analyses and mapping were conducted using R software (version 4.3.2. R Foundation for Statistical Computing, Vienna, Austria, https://cran.r-project.org/).

## Results

### Incidence, death, and DALY of eight bacterial diseases and temporal trend

#### Syphilis

In 2021, the global ASIR for syphilis was 235.47 per 100,000 population (95% UI: 176.40, 307.43 per 100,000 population), with an increase of 8.55% (95% UI: 6.32, 10.79%) compared to 1990. The global age-standardized DALY rate for syphilis was estimated at 107.13 per 100,000 population (95% UI: 41.77, 212.12 per 100,000 population), with a percentage change of −15.10% (95% UI: − 29.51, 0.04%) compared to 1990, which did not show a significant upward or downward trend (*P* > 0.05, equally, Table 1). Syphilis showed an ASMR of 1.19 per 100,000 population (95% UI: 0.47, 2.36 per 100,000 population), declining by − 16.22% (95% UI: − 30.91, − 1.37%) compared to 1990 (Additional file 1: Table S1).

**Table 1:** Age-standardized incidence and DALY rates for syphilis in 1990 and 2021, and the percentage change in the age-standardized rates per 100,000 population by GBD region, from 1990 to 2021

Syphilis	Incidence			DALY		
	Age-standardized incidence rate per 100,000 population (95% UI), 1990	Age-standardized incidence rate per 100,000 population (95% UI), 2021	Percentage change in age-standardized incidence rate, 1990–2021	Age-standardized DALY rate per 100,000 population (95% UI), 1990	Age-standardized DALY rate per 100,000 population (95% UI), 2021	Percentage change in age-standardized DALY rate, 1990–2021
Global	216.93 (163.26, 280.24)	235.47 (176.40, 307.43)	8.55 (6.32, 10.79)	126.18 (48.91, 240.98)	107.13 (41.77, 212.12)	− 15.10 (− 29.51, 0.04)
Male	285.46 (215.57, 369.12)	312.53 (234.16, 406.22)	9.48 (7.73, 11.42)	135.16 (52.42, 263.31)	113.91 (44.38, 226.59)	− 15.73 (− 30.93, 0.44)
Female	146.64 (108.83, 189.80)	157.00 (115.35, 204.53)	7.06 (3.60, 10.24)	116.85 (44.75, 224.11)	99.96 (39.13, 201.43)	− 14.45 (− 28.29, 0.04)
Andean Latin America	247.58 (188.79, 315.86)	234.14 (176.29, 300.45)	− 5.43 (− 8.87, − 2.07)	155.51 (56.68, 321.56)	94.18 (34.14, 200.02)	− 39.44 (− 52.94, − 23.52)
Australasia	90.64 (66.97, 117.78)	86.61 (64.89, 113.11)	− 4.44 (− 8.55, − 0.16)	1.12 (0.85, 1.31)	0.42 (0.34, 0.53)	− 62.48 (− 69.69, − 51.19)
Caribbean	201.68 (155.57, 255.86)	205.67 (156.72, 263.81)	1.98 (− 1.35, 5.20)	163.47 (64.95, 318.72)	157.86 (60.23, 316.41)	− 3.43 (− 29.41, 33.17)
Central Asia	69.34 (53.02, 89.34)	62.51 (47.21, 81.90)	− 9.85 (− 13.20, − 6.63)	12.50 (8.63, 18.51)	7.44 (3.54, 14.31)	− 40.45 (− 62.52, − 14.54)
Central Europe	49.92 (37.06, 65.41)	47.27 (34.83, 61.78)	− 5.31 (− 7.47, − 3.20)	2.80 (1.59, 5.01)	1.61 (0.76, 3.19)	− 42.74 (− 56.09, − 30.44)
Central Latin America	137.31 (103.26, 180.58)	125.20 (93.36, 164.44)	− 8.82 (− 10.61, − 7.14)	10.79 (8.58, 14.00)	5.15 (3.56, 8.11)	− 52.27 (− 63.05, − 38.29)
Central sub-Saharan Africa	1375.45 (1050.64, 1736.19)	1266.92 (956.83, 1630.00)	− 7.89 (− 12.71, − 3.82)	607.12 (233.26, 1214.94)	332.90 (131.12, 647.20)	− 45.17 (− 57.99, − 26.02)
East Asia	148.88 (109.08, 197.99)	145.97 (106.55, 192.75)	− 1.96 (− 3.55, − 0.17)	35.46 (13.50, 72.88)	30.91 (11.54, 67.99)	− 12.83 (− 35.04, 14.82)
Eastern Europe	63.05 (47.77, 81.53)	53.91 (40.30, 70.54)	− 14.51 (− 17.07, − 12.02)	3.87 (3.46, 4.37)	1.45 (1.22, 1.71)	− 62.52 (− 66.53, − 58.27)
Eastern sub-Saharan Africa	975.77 (763.41, 1201.06)	725.34 (552.38, 926.90)	− 25.67 (− 30.27, − 21.52)	495.00 (199.80, 904.26)	283.39 (116.36, 563.39)	− 42.75 (− 55.40, − 27.98)
High-income Asia Pacific	102.15 (76.67, 133.70)	97.95 (72.36, 128.01)	− 4.11 (− 5.92, − 2.10)	10.44 (4.01, 23.27)	6.16 (2.50, 13.33)	− 41.05 (− 60.17, − 10.94)
High-income North America	112.64 (84.21, 147.51)	106.42 (79.20, 138.42)	− 5.52 (− 6.43, − 4.46)	1.61 (1.39, 1.84)	0.78 (0.64, 0.96)	− 51.67 (− 56.43, − 45.91)
North Africa and Middle East	84.89 (62.68, 111.39)	84.35 (62.01, 110.56)	− 0.64 (− 2.45, 1.82)	73.15 (25.82, 151.57)	45.07 (17.11, 94.81)	− 38.39 (− 52.70, − 23.24)
Oceania	277.42 (211.47, 351.49)	253.51 (192.03, 329.96)	− 8.62 (− 14.41, − 3.11)	309.17 (114.33, 620.78)	253.90 (95.02, 491.41)	− 17.88 (− 38.46, 8.98)
South Asia	284.06 (210.06, 370.71)	251.82 (187.19, 328.48)	− 11.35 (− 12.98, − 9.92)	143.65 (55.11, 287.25)	105.39 (40.66, 223.07)	− 26.63 (− 40.29, − 10.97)
Southeast Asia	216.33 (160.94, 283.60)	212.64 (159.40, 277.53)	− 1.71 (− 3.81, 0.09)	83.26 (30.61, 174.26)	78.98 (28.75, 159.52)	− 5.14 (− 23.19, 16.07)
Southern Latin America	192.87 (146.01, 247.24)	186.81 (139.23, 243.29)	− 3.14 (− 8.03, 1.80)	13.43 (10.86, 14.90)	7.29 (4.91, 9.37)	− 45.71 (− 58.16, − 29.28)
Southern sub-Saharan Africa	979.03 (741.12, 1239.20)	623.40 (467.28, 812.80)	− 36.32 (− 40.29, − 32.78)	498.94 (194.36, 942.16)	256.15 (100.69, 533.06)	− 48.66 (− 61.60, − 32.71)
Tropical Latin America	148.32 (108.32, 195.36)	192.44 (146.94, 241.35)	29.75 (19.40, 41.72)	25.68 (18.98, 35.70)	18.12 (12.83, 27.05)	− 29.42 (− 45.96, − 11.17)
Western Europe	74.70 (56.27, 97.67)	71.20 (52.69, 92.95)	− 4.68 (− 7.08, − 2.98)	3.21 (1.94, 5.32)	2.56 (1.08, 5.08)	− 20.21 (− 45.21, 0.81)
Western sub-Saharan Africa	453.01 (342.72, 582.18)	400.61 (299.51, 518.76)	− 11.57 (− 13.51, − 9.66)	242.95 (93.90, 490.63)	152.59 (59.75, 302.96)	− 37.19 (− 47.95, − 25.76)
High SDI	97.62 (73.46, 127.92)	94.64 (70.56, 123.45)	− 3.05 (− 4.09, − 2.00)	6.31 (3.13, 11.98)	4.07 (1.76, 8.20)	− 35.38 (− 48.53, − 20.35)
High-middle SDI	117.31 (87.27, 153.10)	122.16 (90.59, 159.87)	4.13 (2.52, 5.48)	29.63 (12.06, 59.22)	23.60 (9.37, 47.93)	− 20.35 (− 38.80, − 0.07)
Middle SDI	195.34 (144.81, 257.14)	193.33 (143.36, 253.00)	− 1.03 (− 2.59, 0.81)	71.76 (28.22, 143.49)	60.76 (23.27, 121.90)	− 15.33 (− 30.91, 0.78)
Low-middle SDI	290.10 (216.93, 376.48)	271.96 (202.72, 354.69)	− 6.25 (− 7.74, − 4.87)	155.91 (60.23, 302.80)	114.24 (45.04, 229.95)	− 26.73 (− 38.41, − 13.32)
Low SDI	621.28 (479.83, 783.91)	519.69 (395.12, 667.48)	− 16.35 (− 20.09, − 13.54)	339.41 (132.68, 634.01)	203.37 (80.86, 403.18)	− 40.08 (− 51.76, − 25.70)

*DALY* disability-adjusted life year, *GBD* Global Burden of Disease, *SDI* Socio-demographic Index, *UI* uncertainty interval

The ASIR for syphilis was 312.53 per 100,000 population (95% UI: 234.16, 406.22 per 100,000 population) in males in 2021, with an increase of 9.48% (95% UI: 7.73, 11.42%) compared to 1990. The ASIR for syphilis was 157.00 per 100,000 population (95% UI: 115.35, 204.53 per 100,000 population) in females, with an increasing of 7.06% (95% UI: 3.60, 10.24%) compared to 1990. However, age-standardized DALY rate and ASMR of syphilis show no significant differences between male and female individuals in 2021 (*P* > 0.05, equally, Table 1).

#### Gonococcal infection

In 2021, the global ASIR for gonococcal infection was 1096.58 per 100,000 population (95% UI: 838.70, 1385.47 per 100,000 population), with a decline of − 4.24% (95% UI: − 7.45, − 1.00%) compared to 1990. The global age-standardized DALY rate for gonococcal infection was 0.91 per 100,000 population (95% UI: 0.64, 1.29 per 100,000 population), declining by − 13.60% (95% UI: − 21.38, − 6.34%) compared to 1990 (Table 2). The ASMR for gonococcal infection was very low in both 1990 and 2021, with less than 0.10 per 100,000 population regardless of GBD region, gender, and five SDI groups (Additional file 1: Table S2).

**Table 2:** Age-standardized incidence and DALY rates for gonococcal infection in 1990 and 2021, and the percentage change in the age-standardized rates per 100,000 population by GBD region, from 1990 to 2021

Gonococcal infection	Incidence			DALY		
	Age-standardized incidence rate per 100,000 population (95% UI), 1990	Age-standardized incidence rate per 100,000 population (95% UI), 2021	Percentage change in age-standardized incidence rate, 1990–2021	Age-standardized DALY rate per 100,000 population (95% UI), 1990	Age-standardized DALY rate per 100,000 population (95% UI), 2021	Percentage change in age-standardized DALY rate, 1990–2021
Global	1145.19 (869.23, 1488.45)	1096.58 (838.70, 1385.47)	− 4.24 (− 7.45, − 1.00)	1.05 (0.77, 1.45)	0.91 (0.64, 1.29)	− 13.60 (− 21.38, − 6.34)
Male	1444.90 (1097.67, 1865.72)	1451.24 (1114.01, 1839.68)	0.44 (− 2.30, 3.04)	1.25 (0.84, 1.91)	1.15 (0.75, 1.77)	− 8.24 (− 21.60, 0.18)
Female	838.51 (633.10, 1083.56)	732.67 (560.93, 937.39)	− 12.62 (− 17.38, − 7.08)	0.85 (0.64, 1.15)	0.66 (0.49, 0.92)	− 23.14 (− 31.61, − 13.13)
Andean Latin America	233.47 (171.11, 318.28)	231.07 (167.44, 316.86)	− 1.03 (− 6.81, 4.78)	0.65 (0.47, 0.90)	0.52 (0.36, 0.74)	−20.07 (− 30.33, − 8.63)
Australasia	292.85 (228.14, 373.83)	285.24 (223.61, 370.95)	− 2.60 (− 8.76, 3.80)	0.79 (0.49, 1.17)	0.63 (0.39, 0.96)	− 19.50 (− 37.51, 1.42)
Caribbean	1023.01 (728.09, 1468.87)	949.39 (683.99, 1364.70)	− 7.20 (− 11.62, − 2.91)	1.27 (0.96, 1.68)	1.20 (0.91, 1.57)	− 5.60 (− 20.31, 12.47)
Central Asia	2398.00 (1684.60, 3438.05)	2171.74 (1513.97, 3148.68)	− 9.44 (− 13.15, − 5.49)	1.95 (1.48, 2.54)	1.30 (0.86, 1.95)	−33.43 (− 44.19, − 20.48)
Central Europe	1925.92 (1454.58, 2537.43)	1797.22 (1356.13, 2346.85)	− 6.68 (− 9.59, − 3.53)	0.56 (0.38, 0.80)	0.46 (0.29, 0.69)	−18.17 (− 26.22, − 10.64)
Central Latin America	687.64 (525.15, 867.21)	661.55 (508.30, 831.31)	− 3.79 (− 6.61, − 0.91)	0.78 (0.61, 0.99)	0.67 (0.52, 0.89)	− 13.65 (− 20.69, − 5.68)
Central sub-Saharan Africa	1284.54 (923.90, 1773.28)	1250.39 (909.94, 1730.74)	− 2.66 (− 8.98, 3.74)	2.46 (1.44, 3.82)	1.77 (1.16, 2.88)	− 28.23 (− 45.12, − 6.89)
East Asia	1197.46 (817.96, 1716.55)	1058.07 (726.37, 1501.46)	− 11.64 (− 14.50, − 8.91)	0.75 (0.49, 1.14)	0.61 (0.38, 0.94)	− 19.10 (− 29.93, − 8.43)
Eastern Europe	2188.36 (1526.77, 3070.20)	2047.80 (1403.68, 2916.76)	− 6.42 (− 9.21, − 3.48)	0.91 (0.70, 1.22)	0.70 (0.52, 0.98)	− 23.13 (− 29.60, − 16.60)
Eastern sub-Saharan Africa	1439.85 (1105.09, 1889.79)	1384.81 (1066.23, 1800.81)	− 3.82 (− 7.26, − 1.14)	3.23 (1.76, 6.36)	1.88 (1.25, 3.17)	− 41.85 (− 54.12, − 15.39)
High-income Asia Pacific	535.93 (429.85, 661.72)	539.03 (425.71, 666.70)	0.58 (− 3.39, 4.49)	0.56 (0.36, 0.85)	0.46 (0.30, 0.69)	− 17.76 (− 23.49, − 12.06)
High-income North America	351.70 (261.05, 461.15)	356.83 (263.44, 471.46)	1.46 (− 0.98, 3.82)	0.51 (0.36, 0.73)	0.34 (0.24, 0.50)	− 33.29 (− 38.53, − 27.26)
North Africa and Middle East	1262.18 (956.46, 1688.64)	1136.59 (857.25, 1567.48)	− 9.95 (− 14.43, − 5.65)	0.75 (0.50, 1.11)	0.69 (0.46, 1.03)	− 7.76 (− 18.56, 2.75)
Oceania	2449.24 (1791.82, 3308.29)	2641.80 (1918.63, 3654.00)	7.86 (− 3.12, 19.52)	0.82 (0.55, 1.23)	0.70 (0.43, 1.13)	− 14.48 (− 33.16, 8.40)
South Asia	1223.62 (875.48, 1696.57)	1128.48 (804.77, 1588.61)	− 7.78 (− 10.03, − 5.30)	1.70 (1.21, 2.30)	1.20 (0.84, 1.70)	− 29.31 (− 39.99, − 19.38)
Southeast Asia	1271.26 (991.93, 1583.76)	1248.92 (968.34, 1568.38)	− 1.76 (− 5.74, 2.73)	1.00 (0.67, 1.49)	0.95 (0.64, 1.42)	− 4.50 (− 13.65, 6.64)
Southern Latin America	420.40 (316.25, 569.47)	414.71 (309.36, 567.31)	− 1.35 (− 7.00, 4.07)	0.92 (0.67, 1.28)	0.78 (0.56, 1.12)	− 15.43 (− 23.53, − 8.05)
Southern sub-Saharan Africa	4102.37 (3062.04, 5424.95)	3889.32 (2873.26, 5163.10)	− 5.19 (− 8.32, − 1.77)	3.47 (2.54, 4.71)	2.51 (1.74, 3.68)	− 27.57 (− 38.68, − 12.38)
Tropical Latin America	844.38 (575.68, 1211.46)	816.29 (563.35, 1177.63)	− 3.33 (− 6.08, − 0.50)	0.69 (0.53, 0.94)	0.80 (0.61, 1.10)	15.58 (4.81–26.93)
Western Europe	144.08 (115.26, 177.60)	138.84 (111.37, 172.24)	− 3.64 (− 6.37, − 1.00)	0.23 (0.18, 0.30)	0.16 (0.11, 0.23)	− 28.34 (− 37.49, − 19.47)
Western sub-Saharan Africa	1389.59 (1099.14, 1744.08)	1319.91 (1039.54, 1663.52)	− 5.01 (− 7.48, − 2.61)	2.02 (1.23, 3.34)	1.35 (0.90, 2.06)	− 32.89 (− 48.18, − 20.99)
High SDI	493.89 (386.24, 611.10)	480.00 (377.48, 593.37)	− 2.81 (− 4.83, − 0.57)	0.47 (0.34, 0.66)	0.37 (0.25, 0.53)	− 22.15 (− 28.59, − 15.68)
High-middle SDI	1251.94 (942.23, 1638.16)	1131.22 (853.27, 1437.31)	− 9.64 (− 12.76, − 6.33)	0.73 (0.52, 1.03)	0.63 (0.42, 0.91)	− 14.43 (− 22.88, − 4.99)
Middle SDI	1303.80 (953.05, 1768.78)	1209.02 (884.46, 1633.02)	− 7.27 (− 9.33, − 5.23)	1.00 (0.70, 1.43)	0.88 (0.60, 1.31)	− 11.46 (− 18.80, − 4.47)
Low-middle SDI	1209.38 (931.02, 1540.55)	1133.11 (876.41, 1434.11)	− 6.31 (− 8.48, − 4.25)	1.53 (1.12, 2.05)	1.13 (0.82, 1.59)	− 25.69 (− 35.55, − 16.89)
Low SDI	1325.74 (1041.89, 1691.86)	1265.73 (990.00, 1621.98)	− 4.53 (− 7.48, − 1.80)	2.41 (1.48, 3.98)	1.53 (1.06, 2.31)	− 36.56 (− 48.84, − 17.83)

*DALY* disability-adjusted life year, *GB*D Global Burden of Disease, *SDI* Socio-demographic Index, *UI* uncertainty interval

In 2021, the ASIR of gonococcal infection in males was 1451.24 per 100,000 population (95% UI: 1114.01, 1839.68 per 100,000 population) which is significantly higher than that in females (732.67 per 100,000 population, 95% UI: 560.93, 937.39 per 100,000 population, *P* < 0.05). The ASIR of gonococcal infection remained stable in males (percentage change = 0.44, 95% UI: − 2.30, 3.04) and decreased by − 12.62% (95% UI: − 17.38, − 7.08) in females compared to 1990. A similar trend was observed for the age-standardized DALY rates: remained stable in males (percentage change = − 8.24, 95% UI: − 21.60, 0.18) and decreased in females (percentage change = − 23.14, 95% UI: − 31.61, − 13.13, Table 2).

#### Typhoid fever

For typhoid fever, the global ASIR was estimated at 98.56 per 100,000 population (95% UI: 76.64, 126.77 per 100,000 population) in 2021, decreasing by − 69.57% (95% UI: − 72.01, − 66.64%) compared to 1990. The global age-standardized DALY rate was 101.09 per 100,000 population (95% UI: 51.43, 176.36 per 100,000 population), with a decline of − 59.23% (95% UI: − 67.07, − 50.70%) compared to 1990 (Table 3). The global ASMR for typhoid fever was 1.30 per 100,000 population (95% UI: 0.65, 2.24 per 100,000 population) in 2021, decreasing − 59.49% (95% UI: − 66.63, − 51.59%, Additional file 1: Table S3).

**Table 3:** Age-standardized incidence and DALY rates for typhoid fever in 1990 and 2021, and the percentage change in the age-standardized rates per 100,000 population by GBD region, from 1990 to 2021

Typhoid fever	Incidence			DALY		
	Age-standardized incidence rate per 100,000 population (95% UI), 1990	Age-standardized incidence rate per 100,000 population (95% UI), 2021	Percentage change in age-standardized incidence rate, 1990–2021	Age-standardized DALY rate per 100,000 population (95% UI), 1990	Age-standardized DALY rate per 100,000 population (95% UI), 2021	Percentage change in age-standardized DALY rate, 1990–2021
Global	323.86 (252.86, 412.21)	98.56 (76.64, 126.77)	− 69.57 (− 72.01, − 66.64)	247.95 (121.95, 421.12)	101.09 (51.43, 176.36)	− 59.23 (− 67.07, − 50.70)
Male	349.09 (274.47, 443.28)	107.22 (83.70, 137.25)	− 69.29 (− 71.89, − 65.97)	261.61 (128.22, 440.12)	106.19 (53.31, 183.51)	− 59.41 (− 67.17, − 49.75)
Female	298.46 (230.42, 384.34)	89.54 (69.14, 116.33)	− 70.00 (− 72.40, − 67.11)	233.89 (118.26, 397.65)	95.73 (49.06, 168.07)	− 59.07 (− 67.07, − 50.06)
Andean Latin America	2.69 (2.00, 3.73)	1.37 (1.00, 1.90)	− 49.09 (− 55.12, − 43.03)	12.82 (11.32, 14.26)	0.40 (0.20, 0.70)	− 96.92 (− 98.41, − 94.72)
Australasia	0.08 (0.05, 0.13)	0.08 (0.05, 0.13)	− 1.09 (− 21.42, 34.45)	0.00 (0.00, 0.00)	0.00 (0.00, 0.00)	− 10.78 (− 30.24, 17.94)
Caribbean	17.87 (14.39, 22.26)	7.00 (5.58, 8.70)	− 60.79 (− 66.41, − 55.20)	18.95 (8.98, 36.70)	7.27 (3.42, 14.85)	− 61.63 (− 73.07, − 47.34)
Central Asia	1.06 (0.82, 1.32)	0.37 (0.28, 0.48)	− 64.91 (− 69.69, − 59.95)	0.79 (0.53, 1.16)	0.09 (0.05, 0.14)	− 88.64 (− 93.18, − 79.65)
Central Europe	0.20 (0.15, 0.26)	0.13 (0.09, 0.18)	− 34.13 (− 45.33, − 21.43)	0.02 (0.01, 0.02)	0.01 (0.01, 0.01)	− 33.34 (− 46.47, − 19.57)
Central Latin America	39.49 (32.38, 49.66)	12.62 (9.96, 16.45)	− 68.04 (− 71.49, − 65.20)	24.32 (22.43, 26.32)	1.38 (1.15, 1.65)	− 94.34 (− 95.12, − 93.42)
Central sub-Saharan Africa	62.00 (47.70, 82.00)	23.41 (17.74, 30.24)	− 62.24 (− 66.05, − 58.25)	43.37 (20.00, 82.07)	17.72 (8.41, 34.42)	− 59.13 (− 67.70, − 48.97)
East Asia	14.17 (10.91, 18.61)	5.56 (4.37, 7.09)	− 60.74 (− 64.65, − 55.56)	11.75 (5.20, 22.88)	3.51 (1.57, 6.30)	− 70.11 (− 77.30, − 61.07)
Eastern Europe	0.46 (0.34, 0.60)	0.36 (0.27, 0.46)	− 21.93 (− 29.60, − 14.35)	0.11 (0.08, 0.14)	0.02 (0.01, 0.03)	− 82.53 (− 89.61, − 72.70)
Eastern sub-Saharan Africa	403.94 (311.60, 522.32)	115.39 (89.40, 146.73)	− 71.43 (− 72.31, − 70.55)	330.50 (158.42, 592.25)	129.67 (62.38, 232.46)	− 60.77 (− 68.32, − 51.75)
High-income Asia Pacific	0.09 (0.06, 0.12)	0.08 (0.05, 0.11)	− 8.47 (− 22.41, 6.69)	0.02 (0.01, 0.03)	0.01 (0.00, 0.01)	− 64.14 (− 76.41, − 49.49)
High-income North America	0.10 (0.07, 0.14)	0.10 (0.07, 0.14)	− 5.47 (− 14.57, 3.87)	0.01 (0.01, 0.01)	0.01 (0.01, 0.01)	− 9.49 (− 17.25, − 0.56)
North Africa and Middle East	80.44 (62.89, 104.70)	18.95 (14.69, 24.87)	− 76.45 (− 77.70, − 75.21)	62.80 (29.37, 115.78)	15.19 (7.36, 27.55)	− 75.82 (− 80.53, − 71.11)
Oceania	697.97 (516.71, 900.38)	249.90 (186.93, 335.71)	− 64.20 (− 67.44, − 59.30)	522.35 (234.85, 948.78)	254.04 (122.35, 467.12)	− 51.37 (− 61.26, − 35.15)
South Asia	1041.08 (805.17, 1347.56)	269.08 (207.78, 348.25)	− 74.15 (− 76.86, − 70.47)	728.56 (359.52, 1210.76)	260.27 (130.39, 433.25)	− 64.28 (− 72.19, − 54.99)
Southeast Asia	493.96 (385.54, 618.89)	142.59 (110.81, 182.71)	− 71.13 (− 71.98, − 69.96)	358.59 (180.66, 624.09)	133.50 (64.38, 226.23)	− 62.77 (− 70.23, − 54.23)
Southern Latin America	2.17 (1.67, 2.71)	0.66 (0.52, 0.81)	− 69.78 (− 73.36, − 65.69)	3.14 (2.79, 3.49)	0.03 (0.03, 0.04)	− 99.01 (− 99.18, − 98.84)
Southern sub-Saharan Africa	1.60 (1.20, 2.07)	1.34 (1.03, 1.71)	− 15.96 (− 20.60, − 11.75)	1.08 (0.51, 2.07)	1.11 (0.51, 2.09)	2.81 (− 13.55, 24.44)
Tropical Latin America	5.46 (4.13, 7.27)	0.96 (0.72, 1.28)	− 82.50 (− 85.30, − 78.94)	2.06 (1.25, 3.32)	0.16 (0.08, 0.29)	− 92.19 (− 94.37, − 89.83)
Western Europe	0.42 (0.31, 0.59)	0.36 (0.26, 0.50)	− 14.51 (− 19.23, − 8.32)	0.07 (0.06, 0.08)	0.01 (0.01, 0.01)	− 89.55 (− 91.54, − 87.04)
Western sub-Saharan Africa	403.45 (310.73, 519.83)	102.27 (79.97, 130.94)	− 74.65 (− 75.67, − 73.50)	355.95 (164.42, 634.49)	117.45 (55.92, 224.92)	− 67.00 (− 73.31, − 59.51)
High SDI	3.39 (2.59, 4.48)	1.21 (0.98, 1.51)	− 64.35 (− 69.67, − 59.57)	2.11 (0.73, 4.76)	0.52 (0.20, 1.19)	− 75.25 (− 82.60, − 66.13)
High-middle SDI	35.41 (27.64, 45.73)	16.16 (12.52, 20.72)	− 54.38 (− 56.72, − 51.33)	27.11 (13.12, 47.07)	11.42 (5.95, 19.37)	− 57.86 (− 66.42, − 47.48)
Middle SDI	201.12 (159.13, 252.97)	67.52 (52.80, 87.24)	− 66.43 (− 68.79, − 63.80)	146.07 (75.60, 241.61)	58.66 (29.85, 97.40)	− 59.84 (− 66.48, − 52.22)
Low-middle SDI	782.20 (606.64, 1004.27)	191.27 (150.02, 245.12)	− 75.55 (− 77.50, − 72.90)	550.33 (275.24, 911.65)	188.51 (95.99, 318.45)	− 65.75 (− 72.54, − 57.77)
Low SDI	579.16 (449.05, 741.08)	136.37 (106.15, 174.61)	− 76.45 (− 77.91, − 74.61)	444.78 (212.35, 783.26)	139.77 (69.56, 251.34)	− 68.58 (− 74.11, − 61.64)

*DALY* disability-adjusted life year, *GBD* Global Burden of Disease, *SDI* Socio-demographic Index, *UI* uncertainty interval

In 2021, the ASIR of typhoid fever in males and females were 107.22 per 100,000 population (95% UI: 83.70, 137.25 per 100,000 population) and 89.54 per 100,000 population (95% UI: 69.14, 116.33 per 100,000 population), respectively. The age-standardized DALY rates in males and females were 101.09 per 100,000 population (95% UI: 51.43, 176.36 per 100,000 population) and 95.73 per 100,000 population (95% UI: 49.06, 168.07 per 100,000 population), respectively (Table 3).

#### Paratyphoid fever

In 2021, the global ASIR, age-standardized DALY and ASMR for paratyphoid fever were 29.21 per 100,000 population (95% UI: 21.15, 40.28 per 100,000 population), 14.16 per 100,000 population (95% UI: 6.33, 27.37 per 100,000 population), and 0.19 per 100,000 population (95% UI: 0.09, 0.38 per 100,000 population), both declining at least − 72.00% compared to 1990 (Table 4 and Additional file 1: Table S4). The paratyphoid ASMR was very low in 2021, with less than 0.80 per 100,000 population regardless of GBD region, gender, and five SDI groups (Additional file 1: Table S4).

**Table 4:** Age-standardized incidence and DALY rates for paratyphoid fever in 1990 and 2021, and the percentage change in the age-standardized rates per 100,000 population by GBD region, from 1990 to 2021

Paratyphoid fever	Incidence			DALY		
	Age-standardized incidence rate per 100,000 population (95% UI), 1990	Age-standardized incidence rate per 100,000 population (95% UI), 2021	Percentage change in age-standardized incidence rate, 1990–2021	Age-standardized DALY rate per 100,000 population (95% UI), 1990	Age-standardized DALY rate per 100,000 population (95% UI), 2021	Percentage change in age-standardized DALY rate, 1990–2021
Global	137.48 (100.84, 188.01)	29.21 (21.15, 40.28)	− 78.75 (− 81.56, − 76.07)	53.41 (22.41, 107.87)	14.16 (6.33, 27.37)	− 73.48 (− 79.26, − 66.07)
Male	148.53 (108.72, 204.38)	31.44 (22.63, 43.52)	− 78.84 (− 82.00, − 75.57)	56.42 (23.65, 112.67)	14.45 (6.49, 27.88)	− 74.39 (− 79.94, − 66.83)
Female	126.11 (93.10, 174.77)	26.88 (19.21, 36.78)	− 78.68 (− 81.64, − 75.65)	50.29 (20.98, 101.60)	13.87 (6.09, 26.64)	− 72.42 (− 79.53, − 64.50)
Andean Latin America	0.04 (0.03, 0.06)	0.02 (0.01, 0.03)	− 47.95 (− 56.88, − 39.63)	0.02 (0.01, 0.03)	0.02 (0.01, 0.03)	− 3.31 (− 34.99, 48.59)
Australasia	0.07 (0.04, 0.10)	0.10 (0.06, 0.16)	61.43 (26.24, 114.24)	0.00 (0.00, 0.01)	0.00 (0.00, 0.00)	− 73.26 (− 81.14, − 61.16)
Caribbean	1.95 (1.35, 2.77)	2.17 (1.49, 3.05)	11.59 (− 11.17, 40.50)	1.27 (0.46, 2.73)	1.53 (0.54, 3.43)	20.33 (− 19.86, 88.37)
Central Asia	0.02 (0.02, 0.03)	0.01 (0.01, 0.02)	− 40.99 (− 50.52, − 29.71)	0.10 (0.05, 0.19)	0.01 (0.00, 0.01)	− 95.14 (− 97.38, − 90.29)
Central Europe	0.21 (0.16, 0.28)	0.22 (0.16, 0.30)	2.90 (− 18.41, 31.27)	0.03 (0.02, 0.03)	0.01 (0.00, 0.01)	− 80.32 (− 84.05, − 75.94)
Central Latin America	1.45 (1.07, 1.96)	0.52 (0.38, 0.72)	− 64.03 (− 68.80, − 59.72)	0.30 (0.27, 0.36)	0.02 (0.01, 0.02)	− 94.44 (− 95.40, − 93.34)
Central sub-Saharan Africa	0.34 (0.21, 0.51)	0.10 (0.06, 0.15)	− 70.45 (− 76.51, − 63.71)	0.14 (0.05, 0.32)	0.04 (0.02, 0.10)	− 68.48 (− 78.08, − 57.08)
East Asia	8.62 (6.37, 11.55)	3.74 (2.83, 4.85)	− 56.58 (− 61.46, − 49.50)	4.51 (1.71, 9.66)	1.44 (0.58, 2.95)	− 68.12 (− 76.59, − 56.20)
Eastern Europe	0.04 (0.03, 0.06)	0.04 (0.03, 0.05)	− 15.12 (− 23.13, − 7.51)	0.02 (0.01, 0.02)	0.01 (0.01, 0.01)	− 37.99 (− 55.95, − 8.01)
Eastern sub-Saharan Africa	1.98 (1.25, 2.88)	2.44 (1.71, 3.29)	23.50 (− 4.52, 66.58)	0.93 (0.36, 1.97)	1.38 (0.55, 2.77)	48.40 (3.05, 134.73)
High-income Asia Pacific	0.21 (0.15, 0.28)	0.19 (0.13, 0.26)	− 9.38 (− 23.59, 3.78)	0.02 (0.01, 0.02)	0.01 (0.00, 0.01)	− 69.66 (− 76.89, − 58.00)
High-income North America	0.31 (0.23, 0.40)	0.32 (0.24, 0.42)	4.98 (− 2.03, 12.72)	0.02 (0.01, 0.02)	0.03 (0.02, 0.03)	53.60 (36.97, 71.00)
North Africa and Middle East	0.89 (0.61, 1.27)	0.21 (0.14, 0.31)	− 76.01 (− 79.13, − 71.81)	0.39 (0.16, 0.87)	0.10 (0.04, 0.21)	− 75.46 (− 80.88, − 68.82)
Oceania	112.51 (78.96, 153.89)	43.15 (29.97, 60.94)	− 61.64 (− 67.13, − 54.57)	48.19 (18.80, 104.24)	25.18 (9.96, 52.70)	− 47.76 (− 60.07, − 25.91)
South Asia	575.85 (424.39, 787.21)	110.56 (79.47, 150.88)	− 80.80 (− 83.38, − 78.35)	211.43 (88.08, 422.54)	51.64 (23.29, 99.87)	− 75.58 (− 81.02, − 68.48)
Southeast Asia	29.67 (21.42, 39.88)	8.97 (6.47, 12.23)	− 69.77 (− 72.19, − 67.24)	12.26 (5.08, 24.42)	4.59 (1.89, 9.02)	− 62.54 (− 70.18, − 54.74)
Southern Latin America	0.01 (0.01, 0.02)	0.01 (0.00, 0.01)	− 62.07 (− 66.53, − 56.93)	0.01 (0.00, 0.02)	0.01 (0.00, 0.01)	− 30.34 (− 67.12, 51.51)
Southern sub-Saharan Africa	0.09 (0.06, 0.12)	0.10 (0.07, 0.14)	10.16 (− 1.83, 25.63)	0.03 (0.01, 0.08)	0.05 (0.02, 0.10)	33.73 (6.19, 67.44)
Tropical Latin America	0.83 (0.60, 1.15)	0.20 (0.15, 0.27)	− 76.24 (− 79.99, − 71.77)	0.15 (0.08, 0.30)	0.02 (0.01, 0.04)	− 87.02 (− 90.10, − 83.23)
Western Europe	0.21 (0.15, 0.29)	0.28 (0.20, 0.40)	33.75 (22.70, 46.08)	0.01 (0.01, 0.01)	0.01 (0.01, 0.01)	− 29.78 (− 36.30, − 22.90)
Western sub-Saharan Africa	50.61 (36.10, 70.37)	7.01 (4.93, 9.84)	− 86.15 (− 87.89, − 84.06)	25.56 (10.39, 54.40)	5.23 (1.93, 12.01)	− 79.52 (− 85.44, − 73.96)
High SDI	0.50 (0.38, 0.63)	0.33 (0.26, 0.41)	− 33.55 (− 44.45, − 21.69)	0.14 (0.06, 0.28)	0.04 (0.02, 0.06)	− 73.39 (− 79.32, − 61.32)
High-middle SDI	12.12 (8.91, 16.40)	4.33 (3.18, 5.86)	− 64.28 (− 68.16, − 60.54)	5.29 (2.23, 10.16)	1.63 (0.71, 3.15)	− 69.27 (− 76.37, − 56.86)
Middle SDI	66.79 (49.29, 90.57)	17.85 (13.02, 24.48)	− 73.27 (− 76.58, − 69.92)	26.55 (11.37, 52.02)	7.72 (3.35, 14.75)	− 70.92 (− 77.12, − 63.99)
Low-middle SDI	366.53 (271.03, 499.66)	66.83 (48.21, 90.28)	− 81.77 (− 84.22, − 79.52)	130.88 (55.17, 262.58)	30.99 (13.97, 61.16)	− 76.32 (− 81.45, − 69.58)
Low SDI	220.46 (163.20, 296.71)	34.61 (25.32, 45.82)	− 84.30 (− 86.23, − 82.42)	85.52 (33.95, 177.03)	15.78 (6.75, 30.69)	− 81.55 (− 85.69, − 76.36)

*DALY* disability-adjusted life year, *GBD* Global Burden of Disease, *SDI* Socio-demographic Index, *UI* uncertainty interval

In 2021, the paratyphoid ASIR in males and females were 31.44 per 100,000 population (95% UI: 22.63, 43.52 per 100,000 population) and 26.88 per 100,000 population (95% UI: 19.21, 36.78 per 100,000 population), respectively. The age-standardized DALY rates in males and females were 14.45 per 100,000 population (95% UI: 6.49, 27.88 per 100,000 population) and 13.87 per 100,000 population (95% UI: 6.09, 26.64 per 100,000 population), respectively (Table 4).

#### Diphtheria

In 2021, the global ASIR (0.19 per 100,000 population, 95% UI: 0.13, 0.27 per 100,000 population) and ASMR (0.06 per 100,000 population, 95% UI: 0.04, 0.08 per 100,000 population) for diphtheria were both very low. Regardless of the GBD region, gender, or SDI categories, they are below 1.20 per 100,000 population and 0.40 per 100,000 population, respectively. The global age-standardized DALY rate was estimated at 4.75 per 100,000 population (95% UI: 3.19–6.82 per 100,000 population). Compared to 1990, diphtheritia ASIR, age-standardized DALY rate, and ASMR have all decreased by at least − 85.00% (Table 5 and Additional file 1: Table S5).

**Table 5:** Age-standardized incidence and DALY rates for diphtheria in 1990 and 2021, and the percentage change in the age-standardized rates per 100,000 population by GBD region, from 1990 to 2021

Diphtheria	Incidence			DALY		
	Age-standardized incidence rate per 100,000 population (95% UI), 1990	Age-standardized incidence rate per 100,000 population (95% UI), 2021	Percentage change in age-standardized incidence rate, 1990–2021	Age-standardized DALY rate per 100,000 population (95% UI), 1990	Age-standardized DALY rate per 100,000 population (95% UI), 2021	Percentage change in age-standardized DALY rate, 1990–2021
Global	1.45 (1.10, 1.97)	0.19 (0.13, 0.27)	− 86.62 (− 89.95, − 82.52)	35.25 (26.77, 47.85)	4.75 (3.19, 6.82)	− 86.52 (− 90.28, − 81.73)
Male	1.34 (1.00, 1.85)	0.19 (0.13, 0.28)	− 85.72 (− 89.59, − 80.54)	34.84 (25.81, 49.26)	4.99 (3.27, 7.45)	− 85.66 (− 89.92, − 79.62)
Female	1.57 (1.14, 2.17)	0.20 (0.12, 0.29)	− 87.43 (− 91.19, − 82.27)	35.70 (26.71, 48.74)	4.50 (2.87, 6.59)	− 87.40 (− 91.61, − 81.61)
Andean Latin America	0.31 (0.19, 0.48)	0.01 (0.01, 0.01)	− 97.22 (− 98.43, − 94.88)	9.62 (5.70, 15.58)	0.21 (0.14, 0.30)	− 97.80 (− 98.84, − 95.77)
Australasia	0.00 (0.00, 0.00)	0.01 (0.00, 0.02)	1725.60 (802.02, 3408.58)	0.01 (0.00, 0.01)	0.06 (0.03, 0.12)	1104.32 (559.83, 2077.61)
Caribbean	0.66 (0.33, 1.22)	0.15 (0.08, 0.27)	− 76.75 (− 89.55, v48.37)	21.84 (10.39, 39.31)	4.62 (2.17, 8.20)	− 78.84 (− 91.31, − 51.00)
Central Asia	0.16 (0.11, 0.22)	0.01 (0.01, 0.02)	− 92.07 (− 94.95, − 87.41)	1.76 (1.15, 2.57)	0.12 (0.07, 0.17)	− 93.23 (− 96.07, − 87.66)
Central Europe	0.01 (0.01, 0.01)	0.00 (0.00, 0.00)	− 83.84 (− 88.22, − 77.69)	0.07 (0.06, 0.10)	0.01 (0.01, 0.01)	− 88.65 (− 91.77, − 83.79)
Central Latin America	0.04 (0.03, 0.05)	0.00 (0.00, 0.00)	− 96.68 (− 97.83, − 94.98)	0.97 (0.82, 1.16)	0.03 (0.02, 0.04)	− 97.36 (− 98.34, − 95.69)
Central sub-Saharan Africa	5.09 (2.90, 8.06)	0.52 (0.34, 0.78)	− 89.83 (− 94.02, − 82.44)	127.17 (67.36, 216.97)	10.43 (6.37, 16.83)	− 91.80 (− 95.38, − 84.82)
East Asia	0.25 (0.20, 0.31)	0.01 (0.01, 0.01)	− 97.54 (− 98.13, − 96.85)	2.58 (2.11, 3.23)	0.05 (0.04, 0.06)	− 98.24 (− 98.70, − 97.67)
Eastern Europe	0.44 (0.37, 0.54)	0.00 (0.00, 0.01)	− 99.43 (− 99.72, − 99.06)	1.56 (1.35, 1.76)	0.01 (0.01, 0.02)	− 99.36 (− 99.71, − 98.89)
Eastern sub-Saharan Africa	5.13 (3.58, 7.47)	0.51 (0.34, 0.77)	− 90.04 (− 93.39, − 84.85)	127.06 (85.42, 185.43)	10.90 (6.93, 17.05)	− 91.42 (− 94.84, − 85.80)
High-income Asia Pacific	0.01 (0.01, 0.02)	0.00 (0.00, 0.00)	− 83.04 (− 88.73, − 73.27)	0.17 (0.11, 0.26)	0.03 (0.02, 0.04)	− 82.78 (− 89.22, − 70.80)
High-income North America	0.00 (0.00, 0.00)	0.01 (0.00, 0.01)	142.81 (112.12, 173.94)	0.03 (0.02, 0.03)	0.06 (0.05, 0.07)	101.36 (75.36, 127.09)
North Africa and Middle East	1.28 (0.80, 2.02)	0.05 (0.04, 0.08)	− 95.91 (− 97.47, − 93.45)	21.64 (13.40, 34.71)	0.82 (0.54, 1.18)	− 96.21 (− 97.75, − 93.46)
Oceania	1.59 (0.91, 2.52)	1.15 (0.63, 1.83)	− 28.03 (− 61.69, 41.62)	15.20 (8.54, 25.27)	11.15 (5.54, 19.36)	− 26.67 (− 65.39, 55.05)
South Asia	1.59 (1.18, 2.22)	0.04 (0.03, 0.05)	− 97.46 (− 98.13, − 96.50)	42.22 (30.98, 56.85)	0.94 (0.74, 1.18)	− 97.78 (− 98.46, − 96.81)
Southeast Asia	1.33 (1.01, 1.78)	0.09 (0.07, 0.11)	− 93.30 (− 94.92, − 91.19)	14.56 (11.00, 19.46)	0.76 (0.62, 0.93)	− 94.77 (− 96.29, − 92.84)
Southern Latin America	0.02 (0.01, 0.03)	0.00 (0.00, 0.00)	− 98.93 (− 99.29, − 98.46)	0.35 (0.24, 0.49)	0.00 (0.00, 0.00)	− 99.38 (− 99.58, − 99.11)
Southern sub-Saharan Africa	0.20 (0.16, 0.26)	0.10 (0.08, 0.13)	− 50.05 (− 62.89, − 33.70)	3.97 (3.05, 5.12)	1.86 (1.47, 2.42)	− 53.05 (− 66.70, − 32.63)
Tropical Latin America	0.08 (0.06, 0.10)	0.00 (0.00, 0.00)	− 96.04 (− 97.00, − 94.82)	2.63 (2.16, 3.11)	0.09 (0.07, 0.12)	− 96.59 (− 97.54, − 95.38)
Western Europe	0.00 (0.00, 0.00)	0.00 (0.00, 0.00)	− 4.93 (− 20.26, 14.13)	0.01 (0.01, 0.01)	0.01 (0.01, 0.01)	− 21.21 (− 32.88, − 6.56)
Western sub-Saharan Africa	8.04 (5.36, 11.85)	1.01 (0.57, 1.50)	− 87.42 (− 91.69, − 82.14)	215.10 (148.83, 324.24)	26.13 (15.57, 39.15)	− 87.85 (− 92.28, − 82.37)
High SDI	0.01 (0.01, 0.01)	0.00 (0.00, 0.00)	− 54.75 (− 66.09, − 39.73)	0.09 (0.07, 0.13)	0.03 (0.03, 0.04)	− 65.11 (− 76.05, − 51.89)
High-middle SDI	0.17 (0.15, 0.20)	0.00 (0.00, 0.01)	− 97.19 (− 97.67, − 96.57)	1.23 (1.05, 1.50)	0.04 (0.03, 0.05)	− 96.57 (− 97.38, − 95.57)
Middle SDI	0.38 (0.31, 0.47)	0.03 (0.02, 0.03)	− 92.83 (− 94.02, − 91.59)	5.15 (4.35, 6.15)	0.32 (0.27, 0.38)	− 93.74 (− 94.84, − 92.40)
Low-middle SDI	1.67 (1.24, 2.29)	0.07 (0.06, 0.10)	− 95.52 (− 96.61, − 94.02)	38.76 (28.71, 51.93)	1.53 (1.15, 2.03)	− 96.06 (− 97.19, − 94.38)
Low SDI	6.52 (4.78, 9.25)	0.74 (0.49, 1.05)	− 88.67 (− 91.57, − 84.95)	164.77 (120.66, 232.59)	17.72 (11.66, 25.70)	− 89.25 (− 92.28, − 85.27)

*DALY* disability-adjusted life year, *GBD* Global Burden of Disease, *SDI* Socio-demographic Index, *UI* uncertainty interval

In 2021, global diphtheritic ASIR in males (0.19 per 100,000 population, 95% UI: 0.13, 0.28 per 100,000 population) and females (0.20 per 100,000 population, 95% UI: 0.12, 0.29 per 100,000 population) were very close. Similarly, the age-standardized DALY rates in males (4.99 per 100,000 population, 95% UI: 3.27, 7.45 per 100,000 population) and females (4.50 per 100,000 population, 95% UI: 2.87, 6.59 per 100,000 population) were also close (Table 5).

#### Pertussis

In 2021, the global ASIR for pertussis was 110.61 per 100,000 population (95% UI: 78.48, 156.93 per 100,000 population), with a decrease of − 78.90% (95% UI: − 82.85, − 73.62%) compared to 1990. The global age-standardized DALY rate for pertussis was estimated at 70.92 per 100,000 population (95% UI: 31.90, 142.08 per 100,000 population), with a percentage change by − 81.24% (95% UI: − 89.10, − 63.77%) compared to 1990 (Table 6). Pertussis showed an ASMR of 0.81 per 100,000 population (95% UI: 0.36, 1.63 per 100,000 population), declining by − 81.32% (95% UI: − 89.81, − 63.91%) compared to 1990 (Additional file 1: Table S6).

**Table 6:** Age-standardized incidence and DALY rates for pertussis in 1990 and 2021, and the percentage change in the age-standardized rates per 100,000 population by GBD region, from 1990 to 2021

Pertussis	Incidence			DALY		
	Age-standardized incidence rate per 100,000 population (95% UI), 1990	Age-standardized incidence rate per 100,000 population (95% UI), 2021	Percentage change in age-standardized incidence rate, 1990–2021	Age-standardized DALY rate per 100,000 population (95% UI), 1990	Age-standardized DALY rate per 100,000 population (95% UI), 2021	Percentage change in age-standardized DALY rate, 1990–2021
Global	524.20 (399.78, 667.59)	110.61 (78.48, 156.93)	− 78.90 (− 82.85, − 73.62)	378.01 (170.27, 795.04)	70.92 (31.90, 142.08)	− 81.24 (− 89.69, − 64.01)
Male	474.59 (361.95, 604.40)	99.84 (70.76, 141.72)	− 78.96 (− 82.96, − 73.64)	343.51 (151.50, 729.30)	67.21 (31.00, 133.11)	− 80.44 (− 89.10, − 63.77)
Female	576.94 (440.00, 734.77)	122.12 (86.72, 172.80)	− 78.83 (− 82.76, − 73.59)	414.68 (190.39, 872.36)	74.90 (33.09, 148.24)	− 81.94 (− 90.23, − 64.13)
Andean Latin America	485.39 (372.31, 616.20)	142.00 (46.57, 308.15)	− 70.74 (− 89.83, − 35.56)	358.93 (93.13, 919.26)	28.81 (2.07, 113.78)	− 91.97 (− 99.49, − 50.47)
Australasia	192.99 (149.12, 245.89)	1.13 (0.28, 2.58)	− 99.41 (− 99.84, − 98.67)	1.34 (0.79, 2.15)	0.01 (0.00, 0.03)	− 99.06 (− 99.76, − 97.46)
Caribbean	479.82 (367.61, 610.59)	1.94 (0.56, 9.11)	− 99.60 (− 99.86, − 98.00)	449.00 (78.31, 1247.96)	14.02 (0.80, 49.43)	− 96.88 (− 99.83, − 78.52)
Central Asia	374.92 (287.48, 478.09)	20.16 (11.02, 34.69)	− 94.62 (− 96.93, − 91.41)	81.02 (24.13, 196.08)	2.94 (0.67, 8.19)	− 96.37 (− 99.25, − 86.34)
Central Europe	148.81 (115.17, 190.99)	1.86 (0.57, 4.10)	− 98.75 (− 99.56, − 97.35)	19.23 (8.13, 36.48)	0.70 (0.24, 1.41)	− 96.37 (− 99.02, − 89.87)
Central Latin America	531.11 (405.45, 676.52)	42.14 (24.62, 65.87)	− 92.07 (− 95.04, − 88.52)	58.33 (33.29, 102.60)	2.69 (0.95, 7.54)	− 95.39 (− 98.47, − 86.25)
Central sub-Saharan Africa	818.75 (618.84, 1038.28)	475.16 (241.67, 832.71)	− 41.97 (− 68.72, − 6.17)	936.31 (197.88, 2468.61)	323.36 (67.15, 909.88)	− 65.46 (− 91.50, 39.92)
East Asia	286.35 (221.58, 363.47)	17.69 (9.31, 30.44)	− 93.82 (− 96.48, − 90.22)	179.96 (22.12, 524.02)	12.55 (1.42, 34.12)	− 93.03 (− 99.28, − 38.74)
Eastern Europe	543.02 (416.30, 691.22)	13.40 (6.47, 24.24)	− 97.53 (− 98.74, − 95.49)	4.63 (3.09, 6.73)	0.14 (0.06, 0.23)	− 97.06 (− 98.54, − 94.95)
Eastern sub-Saharan Africa	688.05 (521.91, 875.99)	202.54 (131.54, 295.00)	− 70.56 (− 79.30, − 59.90)	839.42 (302.28, 1901.25)	161.28 (59.89, 334.05)	− 80.79 (− 92.34, − 44.42)
High-income Asia Pacific	302.69 (234.72, 383.08)	0.11 (0.02, 0.40)	− 99.96 (− 99.99, − 99.87)	38.24 (6.97, 108.36)	0.03 (0.00, 0.17)	− 99.91 (− 99.99, − 99.08)
High-income North America	69.24 (52.75, 90.12)	20.90 (9.70, 38.45)	− 69.82 (− 85.32, − 46.18)	0.73 (0.51, 1.03)	0.19 (0.08, 0.37)	− 74.46 (− 87.52, − 54.31)
North Africa and Middle East	455.48 (349.41, 577.34)	62.56 (31.06, 114.91)	− 86.26 (− 92.50, − 76.34)	312.36 (136.18, 625.50)	28.86 (9.18, 66.71)	− 90.76 (− 96.80, − 75.79)
Oceania	646.40 (490.53, 823.01)	28.38 (13.64, 49.90)	− 95.61 (− 97.70, − 92.92)	476.05 (110.94, 1264.69)	54.23 (13.29, 154.55)	− 88.61 (− 97.31, − 43.30)
South Asia	749.16 (566.81, 952.12)	131.77 (60.03, 236.13)	− 82.41 (− 91.54, − 70.46)	608.66 (162.03, 1688.97)	66.94 (13.94, 173.26)	− 89.00 (− 97.63, − 51.73)
Southeast Asia	571.48 (436.12, 727.92)	22.69 (13.74, 36.03)	− 96.03 (− 97.33, − 94.43)	374.85 (129.72, 809.89)	12.40 (3.99, 28.30)	− 96.69 (− 98.85, − 90.59)
Southern Latin America	308.57 (238.99, 389.85)	13.29 (5.57, 25.88)	− 95.69 (− 98.11, − 91.82)	8.64 (5.79, 12.10)	0.23 (0.07, 0.55)	− 97.37 (− 99.16, − 94.13)
Southern sub-Saharan Africa	382.52 (295.81, 485.68)	70.84 (28.38, 142.61)	− 81.48 (− 92.22, − 63.98)	232.97 (55.99, 599.43)	32.03 (8.38, 83.29)	− 86.25 (− 96.88, − 45.32)
Tropical Latin America	505.57 (386.92, 643.51)	68.85 (19.73, 154.27)	− 86.38 (− 95.89, − 71.05)	17.12 (8.54, 33.32)	0.85 (0.26, 2.01)	− 95.05 (− 98.59, − 84.30)
Western Europe	255.82 (198.06, 324.48)	4.60 (2.77, 7.20)	− 98.20 (− 98.82, − 97.26)	2.30 (1.52, 3.35)	0.05 (0.03, 0.08)	− 97.97 (− 98.65, − 96.86)
Western sub-Saharan Africa	825.86 (624.59, 1048.72)	223.28 (141.43, 326.30)	− 72.96 (− 80.86, − 63.32)	869.83 (351.21, 1833.15)	181.51 (54.39, 441.91)	− 79.13 (− 92.78, − 46.34)
High SDI	181.33 (140.34, 230.48)	12.29 (7.53, 20.49)	− 93.22 (− 95.55, − 89.58)	14.87 (5.61, 29.03)	0.60 (0.20, 1.40)	− 95.96 (− 98.76, − 88.15)
High-middle SDI	319.86 (247.59, 406.69)	18.65 (12.48, 26.46)	− 94.17 (− 95.61, − 92.47)	95.28 (32.48, 223.82)	5.28 (1.81, 11.12)	− 94.45 (− 98.26, − 80.80)
Middle SDI	433.13 (333.20, 550.16)	59.70 (38.66, 91.79)	− 86.22 (− 90.14, − 81.43)	235.35 (96.38, 510.34)	20.61 (8.48, 41.58)	− 91.24 (− 96.63, − 79.37)
Low-middle SDI	701.89 (531.49, 892.91)	120.77 (77.81, 185.65)	− 82.79 (− 87.72, − 75.78)	546.49 (210.83, 1280.53)	69.17 (26.25, 153.27)	− 87.34 (− 95.03, − 64.92)
Low SDI	836.79 (632.68, 1060.98)	223.73 (164.23, 310.98)	− 73.26 (− 78.15, − 67.41)	909.56 (401.23, 1867.50)	175.43 (72.10, 347.09)	− 80.71 (− 89.51, − 63.90)

*DALY* disability-adjusted life year, *GBD* Global Burden of Disease, *SDI* Socio-demographic Index, *UI* uncertainty interval

The ASIR for pertussis was 99.84 per 100,000 population (95% UI: 70.76, 141.72 per 100,000 population) in males in 2021, with a decline of −78.96% (95% UI: − 82.96, − 73.64%) compared to 1990. The ASIR for pertussis was 122.12 per 100,000 population (95% UI: 86.72, 172.80 per 100,000 population) in females, decreasing by − 78.83% (95% UI: − 82.76, − 73.59%) compared to 1990. However, age-standardized DALY rate and ASMR of pertussis show no significant differences between male and female individuals in 2021 (*P* > 0.05, equally, Table 6).

#### Tetanus

In 2021, the global ASIR (0.69 per 100,000 population, 95% UI: 0.30, 1.09 per 100,000 population) and ASMR (0.29 per 100,000 population, 95% UI: 0.13, 0.45 per 100,000 population) for tetanus were both very low. Without regard to the GBD region, gender, or SDI categories, they are below 3.40 per 100,000 population and 2.00 per 100,000 population, respectively. The global age-standardized DALY rate was estimated at 19.53 per 100,000 population (95% UI: 8.59, 32.34 per 100,000 population). Compared to 1990, tetanus ASIR, age-standardized DALY rate, and ASMR have all decreased by at least −91.00% (Table 7 and Additional file 1: Table S7).

**Table 7:** Age-standardized incidence and DALY rates for tetanus in 1990 and 2021, and the percentage change in the age-standardized rates per 100,000 population by GBD region, from 1990 to 2021

Tetanus	Incidence			DALY		
	Age-standardized incidence rate per 100,000 population (95% UI), 1990	Age-standardized incidence rate per 100,000 population (95% UI), 2021	Percentage change in age-standardized incidence rate, 1990–2021	Age-standardized DALY rate per 100,000 population (95% UI), 1990	Age-standardized DALY rate per 100,000 population (95% UI), 2021	Percentage change in age-standardized DALY rate, 1990–2021
Global	7.90 (4.81, 10.37)	0.69 (0.30, 1.09)	− 91.26 (− 93.91, − 85.83)	264.82 (158.12, 341.51)	19.53 (8.59, 32.34)	− 92.62 (− 95.05, − 87.99)
Male	9.08 (4.58, 12.48)	0.82 (0.33, 1.40)	− 90.97 (− 93.92, − 84.75)	280.08 (143.07, 366.05)	20.71 (8.00, 36.83)	− 92.61 (− 95.55, − 86.99)
Female	6.73 (3.99, 9.28)	0.56 (0.24, 0.92)	− 91.62 (− 94.70, − 85.21)	249.08 (164.87, 343.16)	18.38 (8.38, 31.62)	− 92.62 (− 95.70, − 87.27)
Andean Latin America	1.66 (0.97, 2.24)	0.08 (0.04, 0.12)	− 95.42 (− 96.82, − 92.05)	36.76 (20.04, 53.08)	1.18 (0.63, 1.80)	− 96.78 (− 97.82, − 94.17)
Australasia	0.05 (0.03, 0.07)	0.01 (0.01, 0.02)	− 68.38 (− 80.19, − 52.55)	0.06 (0.04, 0.08)	0.02 (0.01, 0.03)	− 71.96 (− 82.03, − 59.41)
Caribbean	2.24 (0.99, 3.78)	0.36 (0.12, 0.93)	− 83.96 (− 92.87, − 55.80)	65.96 (21.68, 122.06)	8.33 (1.78, 25.61)	− 87.38 (− 95.27, − 62.01)
Central Asia	0.07 (0.05, 0.10)	0.01 (0.01, 0.02)	− 86.22 (− 92.39, − 77.83)	0.76 (0.52, 1.01)	0.08 (0.04, 0.13)	− 89.58 (− 93.94, − 83.58)
Central Europe	0.15 (0.11, 0.20)	0.01 (0.00, 0.01)	− 96.64 (− 97.98, − 95.07)	1.25 (0.95, 1.49)	0.03 (0.02, 0.05)	− 97.56 (− 98.53, − 96.17)
Central Latin America	0.92 (0.76, 1.08)	0.02 (0.01, 0.03)	− 97.60 (− 98.32, − 96.59)	27.43 (23.61, 31.82)	0.48 (0.32, 0.73)	− 98.26 (− 98.79, − 97.54)
Central sub-Saharan Africa	5.15 (1.50, 11.22)	1.27 (0.15, 3.89)	− 75.28 (− 90.81, − 61.35)	165.94 (64.23, 310.36)	36.62 (4.74, 80.62)	− 77.93 (− 93.77, − 62.67)
East Asia	3.39 (1.35, 4.93)	0.06 (0.02, 0.12)	− 98.33 (− 99.06, − 95.90)	84.22 (35.25, 121.09)	0.82 (0.27, 1.75)	− 99.03 (− 99.45, − 97.71)
Eastern Europe	0.11 (0.08, 0.14)	0.00 (0.00, 0.01)	− 97.15 (− 97.94, − 96.30)	1.04 (0.79, 1.25)	0.03 (0.02, 0.04)	− 97.34 (− 97.99, − 96.55)
Eastern sub-Saharan Africa	18.64 (9.49, 28.03)	3.31 (1.41, 5.69)	− 82.26 (− 88.96, − 67.85)	577.03 (315.05, 930.62)	96.46 (44.94, 188.65)	− 83.28 (− 90.19, − 70.78)
High-income Asia Pacific	0.21 (0.15, 0.25)	0.02 (0.01, 0.02)	− 91.76 (− 94.17, − 89.51)	1.40 (0.80, 1.91)	0.05 (0.03, 0.07)	− 96.58 (− 97.81, − 94.61)
High-income North America	0.02 (0.01, 0.02)	0.00 (0.00, 0.00)	− 85.12 (− 88.23, − 82.01)	0.15 (0.12, 0.17)	0.02 (0.01, 0.03)	− 85.63 (− 88.49, − 82.58)
North Africa and Middle East	5.52 (3.35, 8.48)	0.39 (0.12, 0.82)	− 92.96 (− 97.00, − 84.88)	86.23 (51.09, 136.21)	4.76 (1.39, 11.28)	− 94.47 (− 97.63, − 86.24)
Oceania	0.37 (0.09, 1.19)	0.38 (0.06, 1.61)	2.23 (− 56.54, 89.85)	3.13 (0.76, 9.88)	2.76 (0.44, 10.96)	− 12.04 (− 63.02, 66.86)
South Asia	16.94 (9.15, 22.09)	0.87 (0.30, 1.75)	− 94.85 (− 96.93, − 90.41)	648.13 (349.37, 786.68)	25.36 (8.44, 52.19)	− 96.09 (− 97.86, − 91.85)
Southeast Asia	21.54 (11.61, 32.72)	2.14 (1.03, 3.21)	− 90.04 (− 93.35, − 79.78)	386.30 (221.82, 583.13)	25.69 (13.28, 37.92)	− 93.35 (− 95.47, − 85.72)
Southern Latin America	0.61 (0.46, 0.76)	0.02 (0.01, 0.04)	− 96.25 (− 97.49, − 94.38)	3.63 (2.91, 4.34)	0.12 (0.08, 0.20)	− 96.59 (− 97.70, − 94.95)
Southern sub-Saharan Africa	0.68 (0.32, 1.09)	0.31 (0.11, 0.53)	− 54.29 (− 71.76, − 25.72)	11.88 (5.67, 19.13)	4.69 (1.59, 7.83)	− 60.55 (− 77.98, − 34.92)
Tropical Latin America	1.94 (1.58, 2.39)	0.07 (0.05, 0.10)	− 96.37 (− 96.98, − 95.25)	42.47 (36.10, 49.55)	1.02 (0.79, 1.41)	− 97.60 (− 98.05, − 96.92)
Western Europe	0.09 (0.07, 0.11)	0.01 (0.00, 0.01)	− 94.26 (− 95.89, − 92.77)	0.65 (0.53, 0.75)	0.04 (0.03, 0.06)	− 93.40 (− 95.32, − 91.76)
Western sub-Saharan Africa	8.55 (5.04, 14.40)	1.02 (0.48, 1.68)	− 88.03 (− 92.42, − 77.63)	328.63 (192.00, 592.01)	32.79 (17.48, 52.54)	− 90.02 (− 94.02, − 78.70)
High SDI	0.15 (0.10, 0.20)	0.01 (0.01, 0.03)	− 90.83 (− 95.19, − 80.05)	1.55 (0.96, 2.14)	0.09 (0.03, 0.19)	− 94.04 (− 96.97, − 85.75)
High-middle SDI	1.36 (0.53, 2.03)	0.08 (0.02, 0.15)	− 93.86 (− 96.32, − 87.75)	26.79 (9.82, 38.26)	1.13 (0.36, 1.95)	− 95.79 (− 97.25, − 91.42)
Middle SDI	5.46 (3.12, 7.56)	0.45 (0.21, 0.76)	− 91.72 (− 94.37, − 83.70)	123.33 (68.16, 168.01)	6.93 (3.08, 12.01)	− 94.38 (− 96.18, − 88.98)
Low-middle SDI	16.86 (9.36, 22.06)	1.05 (0.40, 1.79)	− 93.77 (− 95.89, − 89.36)	535.31 (280.50, 654.87)	23.27 (8.37, 43.09)	− 95.65 (− 97.32, − 91.47)
Low SDI	17.23 (11.05, 23.55)	1.91 (0.90, 3.12)	− 88.94 (− 92.87, − 83.11)	583.82 (403.24, 827.04)	56.82 (28.84, 93.56)	− 90.27 (− 94.05, − 84.73)

*DALY* disability-adjusted life year, *GBD* Global Burden of Disease, *SDI* Socio-demographic Index, *UI* uncertainty interval

In 2021, global tetanus ASIR in males and females were 0.82 per 100,000 population (95% UI: 0.33, 1.40 per 100,000 population) and 0.56 per 100,000 population (95% UI: 0.24, 0.92 per 100,000 population). The age-standardized DALY rates in males and females were 20.71 per 100,000 population (95% UI: 8.00, 36.83 per 100,000 population) and 18.38 per 100,000 population (95% UI: 8.38, 31.62 per 100,000 population, Table 7).

#### Leprosy

From 1990 to 2021, no estimates of leprosy deaths and ASMR were reported in the database. In 2021, the global ASIR for leprosy was 0.58 per 100,000 population (95% UI: 0.50, 0.68 per 100,000 population), with a decrease of − 64.79% (95% UI: − 65.48, − 64.00%) compared to 1990. The global age-standardized DALY rate for leprosy was 0.25 per 100,000 population (95% UI: 0.17, 0.37 per 100,000 population), declining by − 56.32% (95% UI: − 58.03, − 54.59%) compared to 1990 (Table 8).

**Table 8:** Age-standardized incidence and DALY rates for leprosy in 1990 and 2021, and the percentage change in the age-standardized rates per 100,000 population by GBD region, from 1990 to 2021

Leprosy	Incidence			DALY		
	Age-standardized incidence rate per 100,000 population (95% UI), 1990	Age-standardized incidence rate per 100,000 population (95% UI), 2021	Percentage change in age-standardized incidence rate, 1990–2021	Age-standardized DALY rate per 100,000 population (95% UI), 1990	Age-standardized DALY rate per 100,000 population (95% UI), 2021	Percentage change in age-standardized DALY rate, 1990–2021
Global	1.65 (1.41, 1.92)	0.58 (0.50, 0.68)	− 64.79 (− 65.48, − 64.00)	0.58 (0.38, 0.85)	0.25 (0.17, 0.37)	− 56.32 (− 58.03, − 54.59)
Male	2.22 (1.90, 2.61)	0.76 (0.65, 0.89)	− 65.84 (− 66.54, − 65.07)	0.74 (0.49, 1.10)	0.32 (0.21, 0.47)	− 57.56 (− 59.66, − 55.15)
Female	1.09 (0.92, 1.28)	0.40 (0.34, 0.47)	− 62.93 (− 63.74, − 62.15)	0.43 (0.28, 0.63)	0.19 (0.13, 0.29)	− 54.87 (− 57.23, − 52.64)
Andean Latin America	0.41 (0.36, 0.47)	0.08 (0.07, 0.09)	− 80.25 (− 81.09, − 79.31)	0.21 (0.13, 0.32)	0.04 (0.02, 0.06)	− 81.47 (− 83.47, − 79.40)
Australasia	0.00 (0.00, 0.00)	0.00 (0.00, 0.00)	− 25.27 (− 28.83, − 21.50)	0.00 (0.00, 0.00)	0.00 (0.00, 0.00)	− 23.91 (− 41.91, − 3.43)
Caribbean	0.74 (0.66, 0.82)	0.31 (0.28, 0.33)	− 58.50 (− 60.17, − 6.63)	0.39 (0.25, 0.59)	0.15 (0.10, 0.23)	− 60.48 (− 64.35, − 56.45)
Central Asia	0.25 (0.20, 0.31)	0.14 (0.12, 0.18)	− 41.05 (− 44.26, − 38.02)	0.02 (0.01, 0.04)	0.01 (0.01, 0.02)	− 38.48 (− 42.60, − 34.16)
Central Europe	0.00 (0.00, 0.00)	0.00 (0.00, 0.00)	–	0.00 (0.00, 0.00)	0.00 (0.00, 0.00)	–
Central Latin America	0.36 (0.32, 0.40)	0.13 (0.12, 0.14)	− 63.25 (− 64.21, − 62.21)	0.21 (0.13, 0.31)	0.08 (0.05, 0.11)	− 63.14 (− 66.88, − 59.56)
Central sub-Saharan Africa	6.52 (5.93, 7.11)	1.08 (1.01, 1.15)	− 83.41 (− 84.22, − 82.53)	3.40 (2.22, 4.94)	0.62 (0.39, 0.92)	− 81.75 (− 84.48, − 78.42)
East Asia	0.10 (0.09, 0.12)	0.03 (0.02, 0.04)	− 72.26 (− 73.73, − 70.85)	0.08 (0.05, 0.13)	0.02 (0.01, 0.04)	− 73.33 (− 76.23, − 70.54)
Eastern Europe	0.00 (0.00, 0.00)	0.00 (0.00, 0.00)	–	0.00 (0.00, 0.00)	0.00 (0.00, 0.00)	–
Eastern sub-Saharan Africa	3.29 (2.90, 3.72)	1.03 (0.94, 1.14)	− 68.56 (− 70.00, − 67.01)	1.87 (1.22, 2.68)	0.58 (0.38, 0.84)	− 69.04 (− 71.65, − 65.96)
High-income Asia Pacific	0.02 (0.01, 0.02)	0.01 (0.01, 0.01)	− 55.83 (− 60.06, − 51.87)	0.02 (0.01, 0.03)	0.01 (0.01, 0.01)	− 54.10 (− 60.28, − 47.48)
High-income North America	0.00 (0.00, 0.00)	0.00 (0.00, 0.00)	–	0.00 (0.00, 0.00)	0.00 (0.00, 0.00)	–
North Africa and Middle East	0.30 (0.28, 0.34)	0.09 (0.08, 0.10)	− 71.72 (− 72.58, − 70.83)	0.13 (0.08, 0.19)	0.04 (0.03, 0.06)	− 67.56 (− 70.28, − 64.53)
Oceania	2.87 (2.56, 3.21)	1.61 (1.50, 1.75)	− 43.75 (− 47.30, − 40.44)	1.54 (1.00, 2.22)	0.71 (0.47, 1.04)	− 53.71 (− 60.20, − 46.89)
South Asia	6.31 (5.34, 7.46)	1.32 (1.13, 1.55)	− 79.06 (− 79.55, − 78.45)	2.10 (1.39, 3.08)	0.64 (0.42, 0.95)	− 69.41 (− 71.26, − 67.45)
Southeast Asia	1.72 (1.51, 1.98)	0.88 (0.76, 1.03)	− 48.95 (− 51.05, − 46.83)	0.83 (0.55, 1.22)	0.37 (0.24, 0.55)	− 55.18 (− 58.18, − 51.98)
Southern Latin America	0.24 (0.21, 0.27)	0.10 (0.09, 0.11)	− 57.89 (− 60.49, − 55.15)	0.07 (0.04, 0.11)	0.04 (0.02, 0.06)	− 48.47 (− 51.96, − 44.75)
Southern sub-Saharan Africa	0.09 (0.07, 0.10)	0.06 (0.04, 0.07)	− 38.23 (− 40.08, − 36.32)	0.11 (0.07, 0.17)	0.06 (0.04, 0.10)	− 43.59 (− 46.24, − 40.77)
Tropical Latin America	5.74 (4.76, 6.97)	3.55 (2.97, 4.30)	− 38.12 (− 39.23, − 37.04)	1.85 (1.19, 2.68)	1.35 (0.88, 1.97)	− 26.96 (− 31.06, − 22.37)
Western Europe	0.00 (0.00, 0.00)	0.00 (0.00, 0.00)	–	0.00 (0.00, 0.00)	0.00 (0.00, 0.00)	–
Western sub-Saharan Africa	2.16 (1.93, 2.42)	0.72 (0.64, 0.81)	− 66.80 (− 67.65, − 65.96)	1.11 (0.73, 1.64)	0.40 (0.26, 0.59)	− 63.78 (− 65.46, − 62.02)
High SDI	0.01 (0.01, 0.01)	0.01 (0.01, 0.01)	− 4.17 (− 13.29, 6.76)	0.01 (0.00, 0.01)	0.00 (0.00, 0.01)	− 42.77 (− 47.65, − 38.37)
High-middle SDI	0.13 (0.11, 0.15)	0.08 (0.07, 0.10)	− 37.44 (− 38.84, − 36.05)	0.05 (0.03, 0.08)	0.03 (0.02, 0.05)	− 38.52 (− 42.74, − 33.75)
Middle SDI	1.25 (1.05, 1.50)	0.59 (0.50, 0.70)	− 52.55 (− 53.52, − 51.56)	0.49 (0.32, 0.71)	0.26 (0.17, 0.39)	− 45.98 (− 48.31, − 43.55)
Low-middle SDI	4.66 (3.97, 5.48)	1.17 (1.00, 1.38)	− 74.85 (− 75.39, − 74.23)	1.65 (1.08, 2.41)	0.55 (0.36, 0.80)	− 66.86 (− 68.70, − 65.02)
Low SDI	5.50 (4.87, 6.23)	1.20 (1.07, 1.34)	− 78.25 (− 78.82, − 77.68)	2.24 (1.48, 3.25)	0.64 (0.42, 0.95)	− 71.30 (− 73.12, − 69.24)

*DALY* disability-adjusted life year, *GBD* Global Burden of Disease, *SDI* Socio-demographic Index, *UI* uncertainty interval,—not applicable

In 2021, global ASIR for leprosy in males was 0.76 per 100,000 population (95% UI: 0.65, 0.89 per 100,000 population) which is significantly higher than in that in females (0.40 per 100,000 population, 95% UI: 0.34, 0.47 per 100,000 population, *P* < 0.05). The age-standardized DALY rates in males and females were 0.32 per 100,000 population (95% UI: 0.21, 0.47 per 100,000 population) and 0.19 per 100,000 population (95% UI: 0.13, 0.29 per 100,000 population, Table 8).

### Global distribution of eight bacterial diseases

#### Syphilis

For syphilis in 2021, the ASIR was highest in Central sub-Saharan Africa, followed by Eastern sub-Saharan Africa and Southern sub-Saharan Africa, exceeding 460.00 per 100,000 population (Table 1). The countries with the highest ASMR for syphilis are Equatorial Guinea, Central African Republic, and the Democratic Republic of the Congo. In contrast, the regions with an ASIR below 100.00 per 100,000 population include Central Europe, Central Asia, and Western Europe (Table 1), among which the top three lowest ASIR countries were Bulgaria, Slovakia, and Croatia. The age-standardized DALY rate had a similar geographical distribution, being highest in Central, Eastern and Southern sub-Saharan Africa, greater than 100.00 per 100,000 population (Table 1), and South Sudan, the Central Africa Republic, and Liberia had the dominant DALY rates. In contrast, the age-standardized DALY rate was lowest in high-income North America and Australasia, both below 1.00 per 100,000 population (Additional file 1: Table S9).

#### Gonococcal infection

Gonococcal infection had the highest ASIR in Southern sub-Saharan Africa, followed by Oceania and Central Europe in 2021, exceeding 2170.00 per 100,000 population (Table 2). South Africa, Papua New Guinea and Lesotho were three countries with the highest ASIR. Age-standardized DALY rate was highest in Eastern sub-Saharan Africa, and Central sub-Saharan Africa (Table 2). Somalia, Lesotho, and South Africa were the three age-standardized highest DALY rate countries for gonococcal infection (Additional file 1: Table S9).

#### Typhoid fever

The ASIR of typhoid fever was highest in Oceania, South Asia, and Southeast Asia in 2021, exceeding 142.00 per 100,000 population (Table 3). The age-standardized DALY rate and ASMR had the same trends, which are also dominant in these three regions (Table 3 and Additional file 1: Table S3). Burkina Faso, Bangladesh, and Papua New Guinea were the three most dominant countries with disease incidence burden, compared to Belgium, Croatia and Canada, which were the three least dominant countries (Additional file 1: Table S9).

#### Paratyphoid fever

Oceania and South Asia had the highest ASIR, age-standardized DALY rate and ASMR for paratyphoid fever in 2021, exceeding 112.00 per 100,000 population, 25.00 per 100,000 population and 0.34 per 100,000 population, respectively (Table 4 and Additional file 1: Table S4). For paratyphoid fever, India, Pakistan, and Nepal had the highest ASIR, DALY rate and ASMR (Additional file 1: Table S9).

#### Diphtheria

Diphtheria had the most dominant ASIR in Oceania in 2021, up to 1.15 per 100,000 population (Table 5), but African areas, including Somalia, Central Africa Republic, and Nigeria were the top three ASIR countries. The most dominant age-standardized DALY rate was in Western sub-Saharan Africa, exceeding 215.00 per 100,000 population (Table 5 and Additional file 1: Table S9).

#### Pertussis

For pertussis, the top region of ASIR, age-standardized DALY rate and ASMR was Central sub-Saharan Africa, exceeding 475.00 per 100,000 population, 323.00 per 100,000 population and 3.72 per 100,000 population, respectively in 2021 (Table 6 and Additional file 1: Table S6). Among these regions, Angola, Central African Republic, and Somalia had the highest ASIR (Additional file 1: Table S9).

#### Tetanus

Tetanus had the highest ASIR, age-standardized DALY rate and ASMR in Eastern sub-Saharan Africa in 2021, exceeding 3.31 per 100,000 population, 96.46 per 100,000 population and 1.94 per 100,000 population respectively (Table 7 and Additional file 1: Table S7). Somalia and South Sudan were the two countries with the dominant ASIR and age-standardized DALY rate (Additional file 1: Table S9).

#### Leprosy

Leprosy was the most prevalent in Tropical Latin America, with the highest ASIR and age-standardized DALY rate compared to other regions (Table 8). Micronesia, Kiribati, and Marshall Islands had the highest incidence rate, and Kiribati, Tuvalu and Micronesia had the highest DALY rate. There were no death-related data reported for this disease (Additional file 1: Table S9).

### Gender and age distribution for eight bacterial diseases

**Fig. 1:**
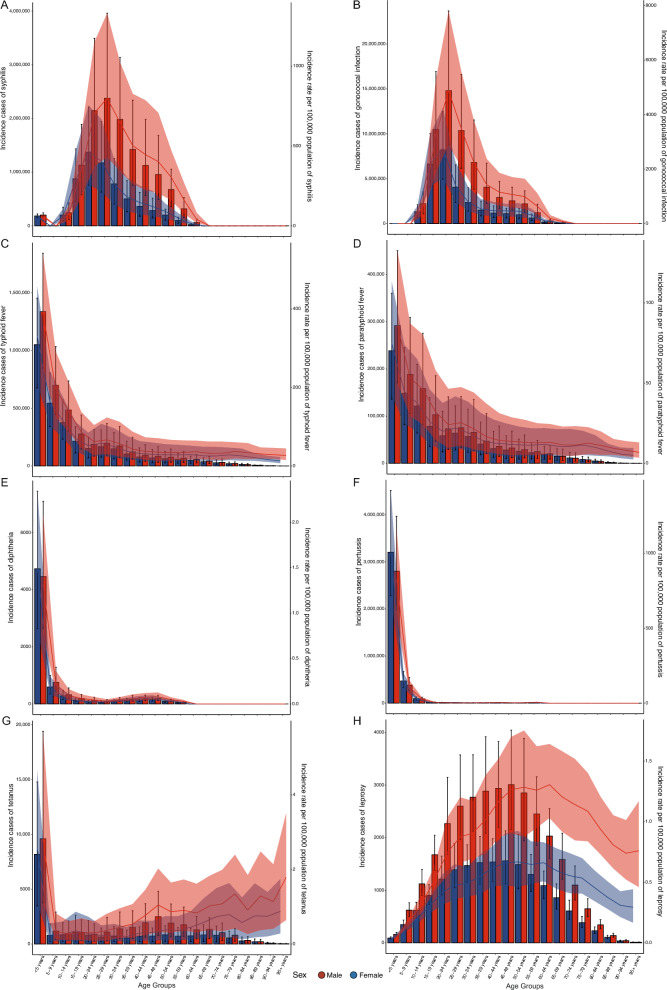
Global number of incidence cases and the point incidence rates per 100,000 population of eight bacterial diseases, by age and sex in 2021. Syphilis (**A**), Gonococcal infection (**B**), Typhoid fever (**C**), Paratyphoid fever (**D**), Diphtheria (**E**), Pertussis (**F**), Tetanus (**G**), Leprosy (**H**)

For syphilis, in the 50–54 age group, the specific incidence rate is higher in males than in females (*P* < 0.05), with no significant differences observed in other age groups (*P* > 0.05, equally). For leprosy, the specific incidence rate between males and females shows no significant difference in the 20–24 and 50–54 age groups (*P* > 0.05, equally), while in other age groups, males have a significantly higher incidence rate than females (*P* < 0.05). The specific incidence rates for gonococcal infection, typhoid and paratyphoid fever, diphtheria, pertussis, and tetanus show no differences between males and females across all age groups (*P* > 0.05, equally, Fig. 1A–H).

In the 45–54, 60–64 age groups and above, the specific DALY rates for syphilis are higher in males than in females (*P* < 0.05), with no significant differences in other age groups (*P* > 0.05, equally). In the 10–24 and 50–54 age groups the specific DALY rates for gonococcal infection are higher in males than in females (*P* < 0.05), with no significant differences in other age groups (*P* > 0.05, equally). For leprosy, only in the > 95 age group, the specific incidence rate is higher in males than in females (*P* < 0.05), with no significant differences observed in other age groups (*P* > 0.05, equally). The specific DALY rate for other five diseases show no differences between males and females across all age groups (*P* > 0.05, equally, Additional file 2: Fig. S1A–H).

In the 45–49, 75–79, 85–89, and > 95 age groups, the specific mortality rate of syphilis is higher in males than in females (*P* < 0.05), with no significant differences in other age groups (*P* > 0.05, equally). The specific mortality rates for other diseases show no differences between males and females across all age groups (*P* > 0.05, equally, Additional file 2: Fig. S2A–G).

The specific DALY rate is highest in the < 5 age group for syphilis, typhoid, paratyphoid, diphtheria, pertussis, and tetanus. The specific incidence rate is highest in the < 5 age group for typhoid, paratyphoid, diphtheria, pertussis, and tetanus (Fig. 1A–H and Additional file 2: Fig. S1A–H).

### Association between eight bacterial diseases burdens and SDI

**Fig. 2:**
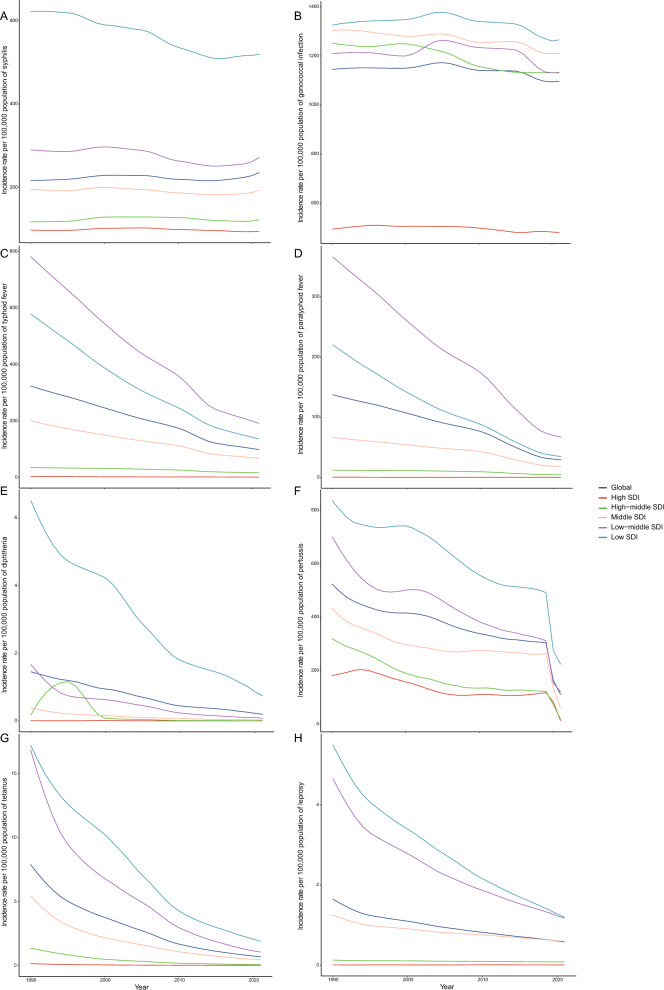
Age-standardized incidence rates per 100,000 population of Syphilis (**A**), Gonococcal infection (**B**), Typhoid fever (**C**), Paratyphoid fever (**D**), Diphtheria (**E**), Pertussis (**F**), Tetanus (**G**) and Leprosy (**H**) from 1990 to 2021 by five SDI regions. *SDI* Socio-demographic Index

Overall, the ASIR and age-standardized DALY rate had a decrease trend from 1990 to 2021 in five SDI regions classification for typhoid fever, paratyphoid fever, diphtheria, pertussis, tetanus, and leprosy globally (Fig. 2C–H and Additional file 2: Fig. S3C–H). From 1990 to 2021, the ASIR for syphilis had a decline trend with increase in the SDI globally. However, syphilis had an increasing ASIR from 2019 (224.31 per 100,000 population, 95% UI: 170.58, 288.58 per 100,000 population) to 2021 (235.47 per 100,000 population, 95% UI: 176.40, 307.63 per 100,000 population) globally (Fig. 2A and Additional file 2: Figs. S3A and S4A). For gonococcal infection, there was a fluctuated trend of ASIR from 1990 to 2021. The global ASIR of gonococcal infection was stable in 2021 (838.70 per 100,000 population, 95% UI: 838.70, 1385.47) compared to 2019 (1096.44 per 100,000 population, 95% UI: 839.31, 1387.21 per 100,000 population), whereas in low SDI regions, there was a slightly increase from 2019 to 2021 (Fig. 2B). For pertussis, a sharp decrease was shown from 2019 to 2021 for ASIR, ASMR and age-standardized DALY rate: for ASIR, from 304.50 per 100,000 population (95% UI: 235.39, 387.54 per 100,000 population) to 110.61 per 100,000 population (95% UI: 78.48, 156.93 per 100,000 population); for age-standardized DALY rate, from 179.72 per 100,000 population (95% UI: 86.16, 309.62 per 100,000 population) to 70.92 per 100,000 population (95% UI: 31.90, 142.08 per 100,000 population); for ASMR from 2.04 per 100,000 population (95% UI: 0.98, 3.53 per 100,000 population) to 0.81 per 100,000 population (95% UI: 0.36, 1.63 per 100,000 population, Fig. 2F and Additional file 2: Figs. S3F and S4F).

In 2021, ASIR of syphilis and pertussis showed a sharp decline with SDI increased, and typhoid fever and paratyphoid fever, diphtheria, tetanus, and leprosy had a slight decreasing trend with increasing SDI. ASIR of gonococcal infection kept stable first and then decreased with increasing SDI (Additional file 2: Fig. S5). There was a sharp decrease with rising SDI for age-standardized DALY rate in syphilis, gonococcal infection, pertussis, and leprosy, whereas there was a slight decrease for typhoid fever, paratyphoid fever, diphtheria, and tetanus (Additional file 2: Fig. S6). The ASMR of syphilis, gonococcal infection, and pertussis had a steeply inverse relationship with SDI, whereas typhoid fever, paratyphoid fever, diphtheria, and tetanus had a slightly negative correlation with SDI (Additional file 2: Fig. S7).

From 1990 to 2021, the ASIR, age-standardized DALY rate and ASMR for syphilis, diphtheria, pertussis, and tetanus declined rapidly with increases in SDI in 21 GBD regions globally (Additional file 2: Fig. S8, AEFG & S9, AEFG & S10, AEFG). However, these trends varied across different GBD regions. The ASIR, ASMR and age-standardized DALY rate for syphilis increased and then decreased with rising SDI in Central sub Saharan Africa, whereas was stable in North Africa and Middle East, Central Europe, high-income Asia Pacific, and Eastern Europe (Additional file 2: Figs. S8A, S9A and S10A). The ASIR of gonococcal infection was relatively stable in all GBD regions except for Southern sub-Saharan Africa and Oceania (increased and then decreased with increasing SDI). By contrast, age-standardized DALY rate and ASMR of gonococcal infection decreased globally with rising SDI (Additional file 2: Fig. S8B, S9B and S10B). The ASIR, ASMR, and age-standardized DALY rates for typhoid fever and paratyphoid fever declined sharply with increasing SDI in South Asia, Southeast Asia, and Central sub-Saharan Africa (Additional file 2: Figs. S8CD, S9CD and S10CD). The ASIR, age-standardized DALY rate and ASMR for diphtheria decreased with rising SDI in Western sub-Saharan Africa, South Asia, and Southeast Asia, whereas East Asia, Western Europe and Australasia kept stable with rising SDI (Additional file 2: Figs. S8E, S9E and S10E). The ASIR of pertussis in high-income Asia Pacific decreased with rising SDI, whereas the age-standardized DALY rate of pertussis in Caribbean declined swiftly with increasing SDI, reached a bottom, and then increased sharply with further increases in SDI (Additional file 2: Figs. S8F and S9F). The ASIR, age-standardized DALY rate and ASMR of tetanus in South Asia, Southeast Asia, Central sub-Saharan Africa decreased sharply (Additional file 2: Figs. S8G, S9G and S10G). The ASIR and age-standardized DALY rate of leprosy in South Asia and Central sub-Saharan Africa decreased with rising SDI, while North Africa and Middle East kept stable with rising SDI (Additional file 2: Figs. S8H and S9H).

### Variance analysis in 21 geographic regions of the globe

**Fig. 3:**
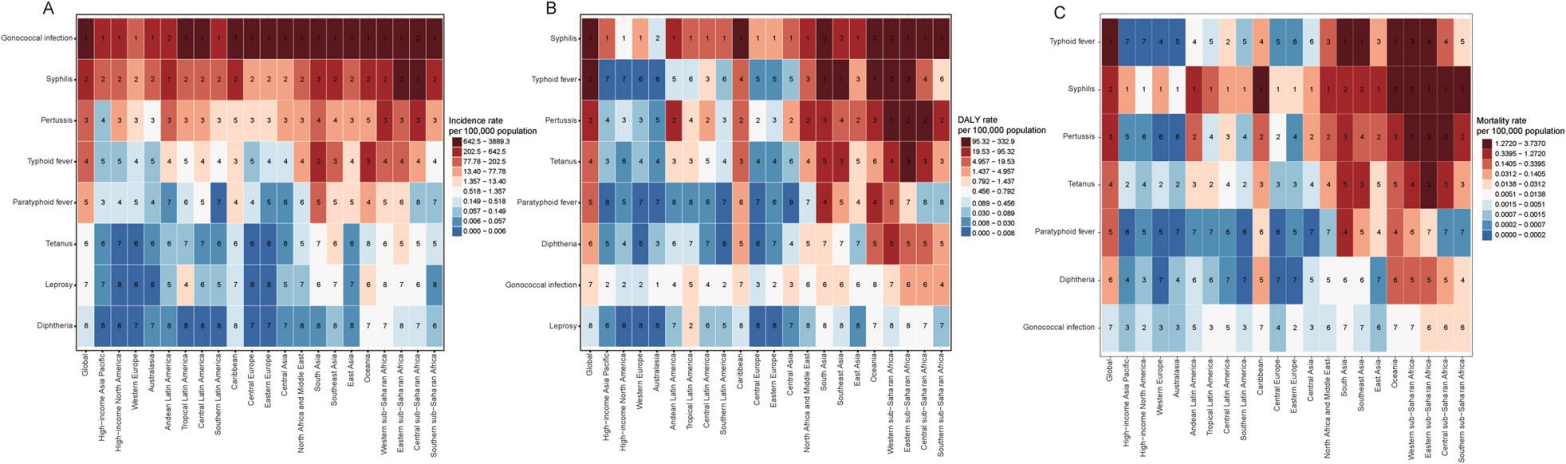
Heatmap of eight specific bacterial infections in 21 GBD regions in 2021. Age-standardized incidence rate (**A**), age-standardized DALY rate (**B**), and age-standardized mortality rate per 100,000 population (**C**). DALY: disability-adjusted life year, *GBD* Global Burden of Disease

In 2021, globally, among the eight bacterial infections, the top three diseases with the highest ASIR were gonorrhea, syphilis, and pertussis; the top three diseases with the highest age-standardized DALY rate were syphilis, typhoid, and pertussis; and the top three diseases with the highest ASMR were typhoid, syphilis, and pertussis (Fig. 3A–C). The ASIR of typhoid fever was notably higher in South Asia compared to other GBD regions. Typhoid and paratyphoid fever showed relatively higher DALY rates in South Asia and Oceania. (Fig. 3A–B).

## Discussion

This study provides a comprehensive estimation of the global incidence, DALYs, and death of eight bacterial diseases. In 2021, except for the two STIs, syphilis and gonococcal infections, the age-standardized DALY rates for all other diseases declined by at least 55% compared to 1990. Compared to 2019, when the COVID-19 pandemic began, the global ASIR and age-standardized DALY rate of syphilis increased in 2021, gonococcal infection rates remained stable, and the other infectious diseases’ rates decreased, with pertussis showing a particularly steep reduction. Regions with higher SDI experienced a lower burden of these eight diseases, whereas low SDI regions continue to require considerable attention for future prevention and control efforts.

### Diphtheria, pertussis and tetanus

The ASIR and age-standardized DALY rate for diphtheria, pertussis, and tetanus saw a notable reduction, owing to the widespread adoption of the DTP vaccine [24]. These diseases exhibited a notably higher age-standardized DALY rate among children under 5 years old, especially in regions with a comparatively low SDI such as sub-Saharan African areas. In such areas, healthcare systems are relatively underdeveloped, and opportunities for bacteria transmission are higher [25]. Additionally, the DTP vaccine requires three doses to produce protective antibodies, and complete vaccination has not been achieved in these areas [26, 27]. Therefore, to better control these three infections, DTP vaccine penetration in the impoverished areas needs to be strengthened. Notably, there was a steep decrease in pertussis cases between 2019 and 2021, possibly owing to the protective effects of COVID-19 quarantine measures. The related measures including social isolation, wearing masks, maintaining hand hygiene, and limiting public gatherings, have effectively mitigated the spread of not only SARS-CoV-2 but also other respiratory infections, including pertussis [28].

### Syphilis and gonococcal infection

While high SDI areas have better prevention and control measures for STIs compared to low SDI areas, syphilis, and gonococcal infection remain problematic even in high SDI regions. The higher age-standardized DALY rates for gonococcal infection may be attributed to some youth having multiple and occasional sexual partners, as well as the increasing resistance of *Neisseria gonorrhoeae* to many commonly used antibiotics, which complicates treatment [29, 30]. Syphilis contributes to increased age-standardized DALY rates in newborns, primarily due to congenital syphilis, which is transmitted from infected mothers to their fetuses, causing serious health problems and a substantial mortality rate [31]. Since 2020, the ASIR and age-standardized DALY rates of syphilis have increased in many regions, which is likely due to the redirection of medical attention towards the COVID-19 pandemic, impeding access to testing and treatment for pregnant women [32, 33].

### Typhoid and paratyphoid fevers

South Asia continues to be a significant epicenter for typhoid fever, as was the case in 2021 GBD study [34]. Despite a continued decline in ASIR of typhoid and paratyphoid fevers in South Asia, the disease burden remains considerable. Water, sanitation, and hygiene (WASH) are critically important in controlling enteric fever, but improving these aspects is usually a long process. In the short term, reducing the disease burden of typhoid and paratyphoid fevers in South Asia requires immunization coverage. Some countries have already started immunization programs for typhoid fever, but eliminating typhoid fever transmission in the region requires more countries to implement vaccination strategies [35, 36].

### Leprosy

Leprosy, classified as a neglected tropical disease, exhibits the lowest disease burden among the range of bacterial infections under study. However, it has great damage to peripheral nerves, resulting in loss of sensation, resulting in skin damage and disability [37]. Leprosy-related deaths are mainly due to complications and chronic conditions associated with the disease [38]. Tropical areas, such as tropical Latin America, have relatively higher leprosy ASIR and age-standardized DALY rates. Vaccination has proven effective in preventing and controlling leprosy before it manifests [39]. The male group exhibited a higher DALY rate than females, which is probably due to greater exposure of men to the *Mycobacterium leprae* bacillus or the lack of full medical examinations for women in some cultures [40].

One Health is an increasingly prominent approach that emphasizes the interconnectedness of human, animal, and environmental health. This holistic strategy acknowledges that human death is closely linked to the health of animals and our shared environment. As global challenges such as antimicrobial resistance, zoonotic diseases, and climate change intensify, the One Health approach has gained significant attention and importance [41]. To address the ongoing threat of bacterial infectious diseases, a One Health strategy that integrates robust policy support, multi-sectoral collaboration, animal health monitoring, environmental management, and enhanced research is crucial for effective and comprehensive bacterial infection control [42]. For typhoid and paratyphoid fever, prevention involves improving WASH alongside effective vaccination campaigns. Environmental management is essential to reduce contamination sources, while public health education raises awareness about hygiene practices [43]. Evidence suggests that armadillos can harbor the *Mycobacterium leprae* that cause leprosy [44]. Monitoring and managing these animal reservoirs, along with studying the One Health microbiome across humans, animals, and the environment, are crucial to prevent zoonotic transmission and control the disease [45].

This study has several limitations. First, significant variations in the quality and availability of data from GBD databases can occur across different regions and countries. In many low- and middle-income countries, limited health data and incomplete reporting can lead to uncertainties in the estimates. Second, while useful, the SDI may not fully capture the complex interplay of social, economic, and environmental factors influencing disease burden. Finally, because GBD 2021 data are model-based rather than derived from real-world data, they may lead to potential overestimation or underestimation.

## Conclusions

This study utilized current data from the GBD 2021 to estimate the disease burdens of syphilis, gonococcal infection, typhoid and paratyphoid fever, diphtheria, pertussis, tetanus, and leprosy. Despite an overall decline in eight diseases, it remains a significant challenge to global public health, especially in low SDI areas. To further alleviate burden of disease on society, proactive intervention strategies at governmental and medical levels should be implemented based on the geographical and epidemiological distribution, including multi-sectoral collaboration, policy support, WASH management, and enhanced research.

## Supplementary Information

Additional file 1: Table S1 Incident cases, DALYs, deaths and age-standardized mortality rates for syphilis in 1990 and 2021, and the percentage change in the age-standardized mortality rates per 100,000 population by GBD region, from 1990 to 2021. Table S2 Incident cases, DALYs, deaths and age-standardized mortality rates for gonococcal infection in 1990 and 2021, and the percentage change in the age-standardized mortality rates per 100,000 population by GBD region, from 1990 to 2021. Table S3 Incident cases, DALYs, deaths and age-standardized mortality rates for typhoid fever in 1990 and 2021, and the percentage change in the age-standardized mortality rates per 100,000 population by GBD region, from 1990 to 2021. Table S4 Incident cases, DALYs, deaths and age-standardized mortality rates for paratyphoid fever in 1990 and 2021, and the percentage change in the age-standardized mortality rates per 100,000 population by GBD region, from 1990 to 2021. Table S5 Incident cases, DALYs, deaths and age-standardized mortality rates for diphtheria in 1990 and 2021, and the percentage change in the age-standardized mortality rates per 100,000 population by GBD region, from 1990 to 2021. Table S6 Incident cases, DALYs, deaths and age-standardized mortality rates for pertussis in 1990 and 2021, and the percentage change in the age-standardized mortality rates per 100,000 population by GBD region, from 1990 to 2021. Table S7 Incident cases, DALYs, deaths and age-standardized mortality rates for tetanus in 1990 and 2021, and the percentage change in the age-standardized mortality rates per 100,000 population by GBD region, from 1990 to 2021. Table S8 Incident cases and DALYs for leprosy in 1990 and 2021 by GBD region. Table S9 Age-standardized rate of eight diseases in 2021 for 204 countries and territories for both sexes population (the top three and the bottom three).

Additional file 2: Fig. S1 Global number of DALYs and the point DALY rates per 100,000 population of eight bacterial disease, by age and sex in 2021. Fig. S2 Global number of death cases and the point mortality rates per 100,000 population of eight bacterial diseases, by age and sex in 2021. Fig. S3 Age-standardized DALY rates per 100,000 population of eight bacterial diseases from 1990 to 2021 by five SDI regions. Fig. S4 Age-standardized mortality rates per 100,000 population of eight bacterial diseases from 1990 to 2021 by five SDI regions. Fig. S5 Relationship between SDI and age-standardized incidence rates of eight bacterial diseases by country in 2021. Fig. S6 Relationship between SDI and age-standardized DALY rates of eight bacterial diseases by country in 2021. Fig. S7 Relationship between SDI and age-standardized mortality rates of eight bacterial diseases by country in 2021. Fig. S8 Age-standardized incidence rates per 100,000 population of eight bacterial diseases for the 21 GBD regions by SDI, 1990–2021. Fig. S9 Age-standardized DALY rates per 100,000 population of eight bacterial diseases for the 21 GBD regions by SDI, 1990–2021. Fig. S10 Age-standardized mortality rates per 100,000 population of eight bacterial diseases for the 21 GBD regions by SDI, 1990–2021.

## Data Availability

The data used were publicly for this study. The website of the data is https://vizhub.healthdata.org/gbd-results/.

## Abbreviations

ASIR: Age-standardized incidence rate
ASMR: Age-standardized mortality rate
ASR: Age-standardized rate
COVID-19: Coronavirus disease 2019
DALY: Disability-adjusted life year
DTP: Diphtheria-tetanus-pertussis
GBD: Global Burden of Disease
HIV: Human immunodeficiency virus
ICD: International Classification of Diseases
IHME: Institute for Health Metrics and Evaluation
SDI: Socio-demographic Index
STI: Sexually transmitted infection
TB: Tuberculosis
UI: Uncertainty interval
WASH: Water, sanitation, and hygiene
WHO: World Health Organization
