# Supplementary material for: Global patterns of syphilis, gonococcal infection, typhoid fever, paratyphoid fever, diphtheria, pertussis, tetanus, and leprosy from 1990 to 2021: findings from the Global Burden of Disease Study 2021
Source: Infect Dis Poverty. 2024 Sep 13;13:66. doi: 10.1186/s40249-024-01231-2 (PMC11396325; doi:10.1186/s40249-024-01231-2)
Supplement: Supplementary file 2 — Additional file 2: Fig. S1 Global number of DALYs and the point DALY rates per 100,000 population of eight bacterial disease, by age and sex in 2021. Fig. S2 Global number of death cases and the point mortality rates per 100,000 population of eight bacterial diseases, by age and sex in 2021. Fig. S3 Age-standardized DALY rates per 100,000 population of eight bacterial diseases from 1990 to 2021 by five SDI regions. Fig. S4 Age-standardized mortality rates per 100,000 population of eight bacterial diseases from 1990 to 2021 by five SDI regions. Fig. S5 Relationship between SDI and age-standardized incidence rates of eight bacterial diseases by country in 2021. Fig. S6 Relationship between SDI and age-standardized DALY rates of eight bacterial diseases by country in 2021. Fig. S7 Relationship between SDI and age-standardized mortality rates of eight bacterial diseases by country in 2021. Fig. S8 Age-standardized incidence rates per 100,000 population of eight bacterial diseases for the 21 GBD regions by SDI, 1990–2021. Fig. S9 Age-standardized DALY rates per 100,000 population of eight bacterial diseases for the 21 GBD regions by SDI, 1990–2021. Fig. S10 Age-standardized mortality rates per 100,000 population of eight bacterial diseases for the 21 GBD regions by SDI, 1990–2021. [file 40249_2024_1231_MOESM2_ESM.docx]

Global patterns of syphilis, gonococcal infection, typhoid fever, paratyphoid fever, diphtheria, pertussis, tetanus, and leprosy from 1990 to 2021: findings from the Global Burden of Disease Study 2021

Weiye Chen^1,2,3†^, Yiming Chen^1,2,3†^, Zile Cheng^1,2,3^, Yiwen Chen^1,2,3^, Chao Lv^1,2,3^, Lingchao Ma^1,2,3^, Nan Zhou^1,2,3^, Jing Qian^1,2,3^, Chang Liu^4^, Min Li^1,3*^, Xiaokui Guo^1,3*^, and Yongzhang Zhu^1,3*^

^1^ School of Global Health, Chinese Center for Tropical Diseases Research, Shanghai Jiao Tong University School of Medicine, Shanghai, China

^2^ School of Public Health, Shanghai Jiao Tong University School of Medicine, Shanghai, China

^3^ Key Laboratory of Parasite and Vector Biology, National Institute of Parasitic Diseases, Chinese Center for Disease Control and Prevention (Chinese Center for Tropical Diseases Research), Shanghai 200025, China

^4^ Department of Immunology and Microbiology, Shanghai Jiao Tong University School of Medicine, Shanghai, China

^†^Weiye Chen and Yiming Chen contributed equally to this work.

^*^Correspondence:

Yongzhang Zhu

[yzhzhu@hotmail.com](mailto:yzhzhu@hotmail.com)

Xiaokui Guo

[xkguo@shsmu.edu.cn](mailto:xkguo@shsmu.edu.cn)

Min Li

[minli@shsmu.edu.cn](mailto:minli@shsmu.edu.cn)

1 Fig. S1 Global number of DALYs and the point DALY rate per 100,000 population of eight bacterial diseases, by age and sex in 2021.

2 Fig. S2 Global number of death cases and the point death rate per 100,000 population of eight bacterial diseases, by age and sex in 2021.

3 Fig. S3 Age-standardized DALY rates per 100,000 population of eight bacterial diseases from 1990 to 2021 by five SDI regions.

4 Fig. S4 Age-standardized mortality rates per 100,000 population of eight bacterial diseases from 1990 to 2021 by five SDI regions.

5 Fig. S5 Relationship between SDI and age-standardized incidence rates of eight bacterial diseases by country in 2021.

6 Fig. S6 Relationship between SDI and age-standardized DALY rates of eight bacterial diseases by country in 2021.

7 Fig. S7 Relationship between SDI and age-standardized mortality rates of eight bacterial diseases by country in 2021.

8 Fig. S8 Age-standardized incidence rates per 100,000 population of eight bacterial diseases for the 21 GBD regions by SDI, 1990-2021.

9 Fig. S9 Age-standardized DALY rates per 100,000 population of eight bacterial diseases for the 21 GBD regions by SDI, 1990-2021.

10 Fig. S10 Age-standardized mortality rates per 100,000 population of eight bacterial diseases for the 21 GBD regions by SDI, 1990-2021.

**
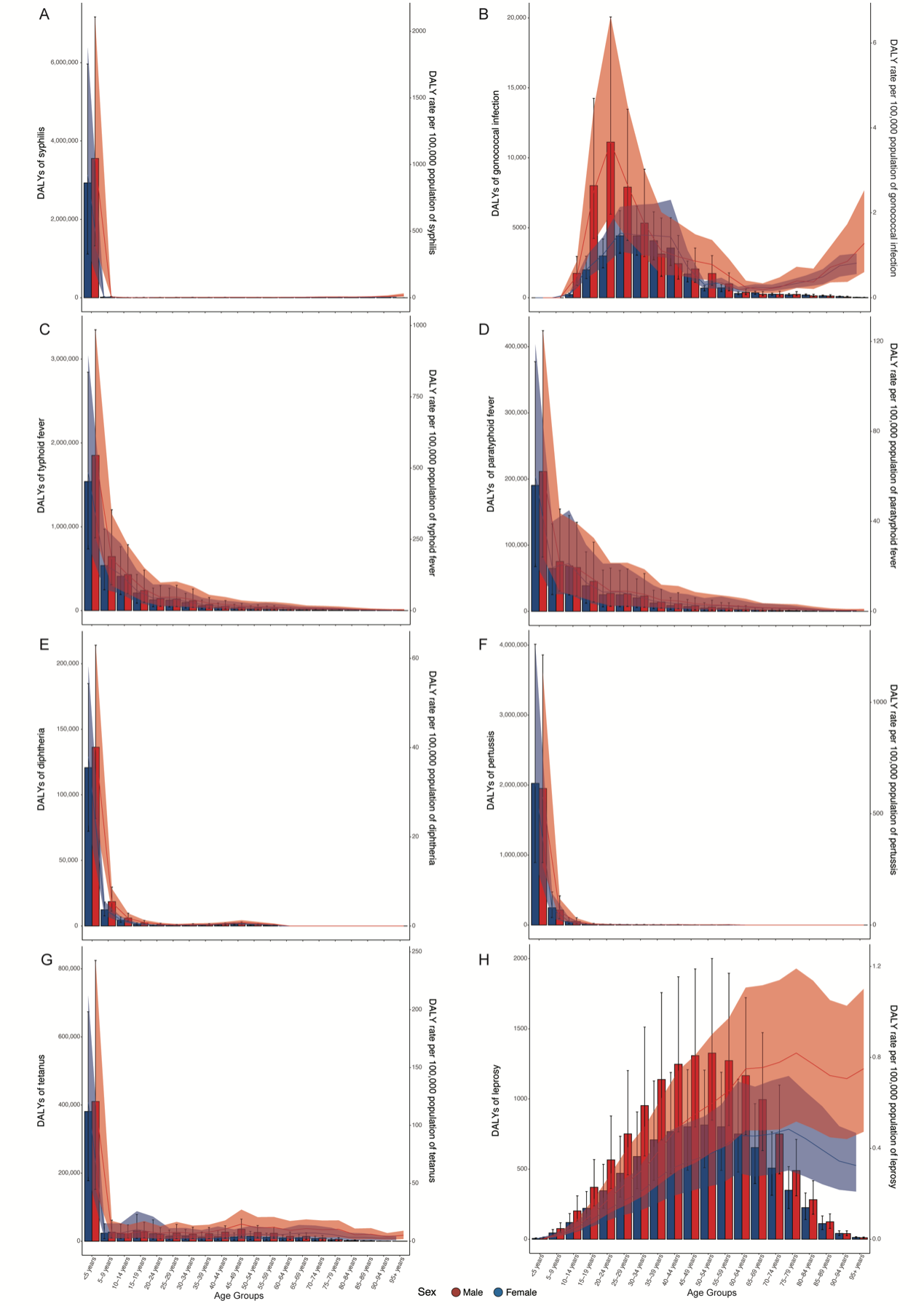
**

Fig. S1 Global number of DALYs and the point DALY rates per 100,000 population of eight bacterial diseases, by age and sex in 2021; Syphilis (A), Gonococcal infection (B), Typhoid fever (C), Paratyphoid fever (D), Diphtheria (E), Pertussis (F), Tetanus (G), Leprosy (H). (Abbreviation: DALY: disability-adjusted life year)


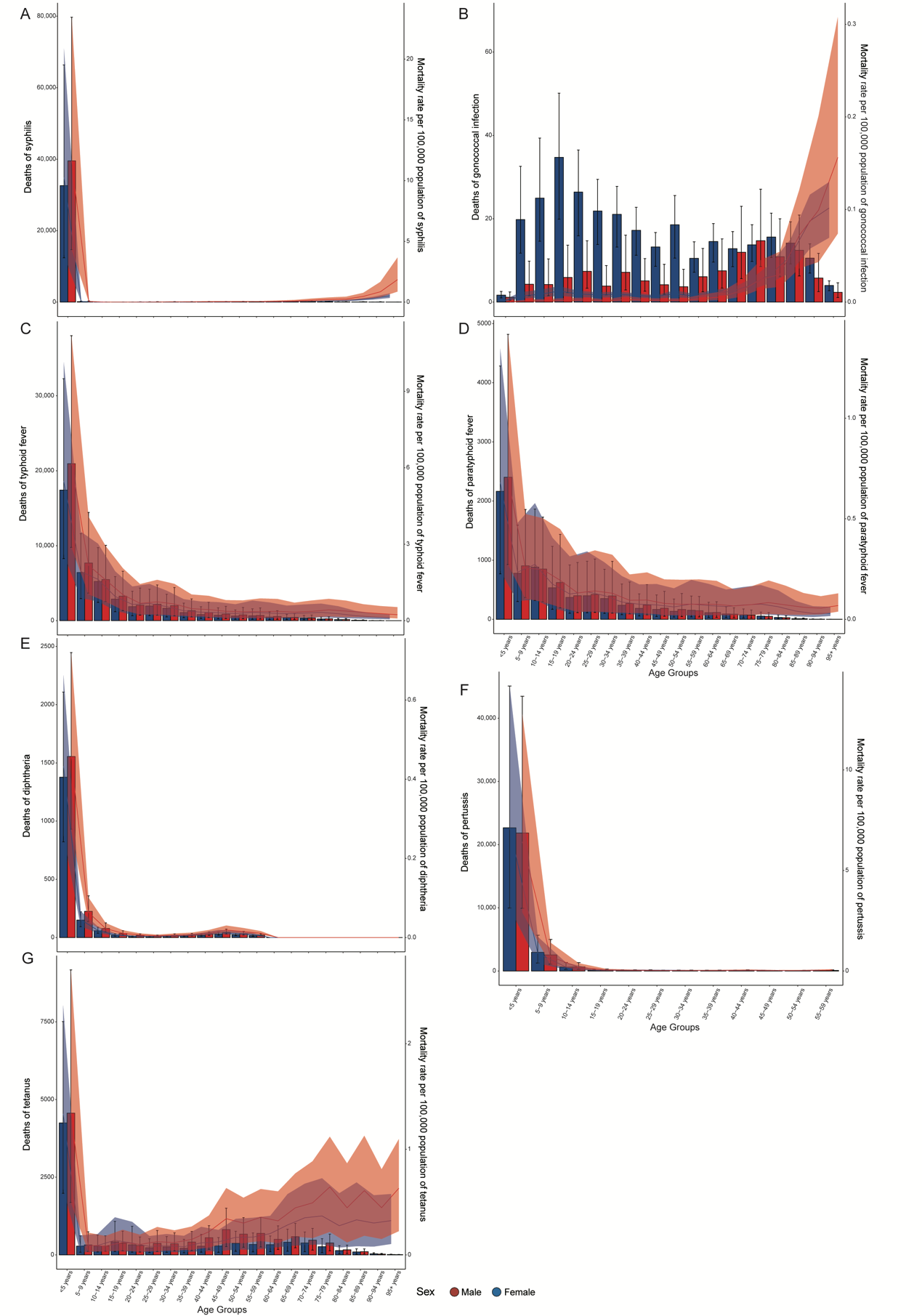


Fig. S2 Global number of death cases and the point death rates per 100,000 population of eight bacterial diseases, by age and sex in 2021; Syphilis (A), Gonococcal infection (B), Typhoid fever (C), Paratyphoid fever (D), Diphtheria (E), Pertussis (F), Tetanus (G).

**
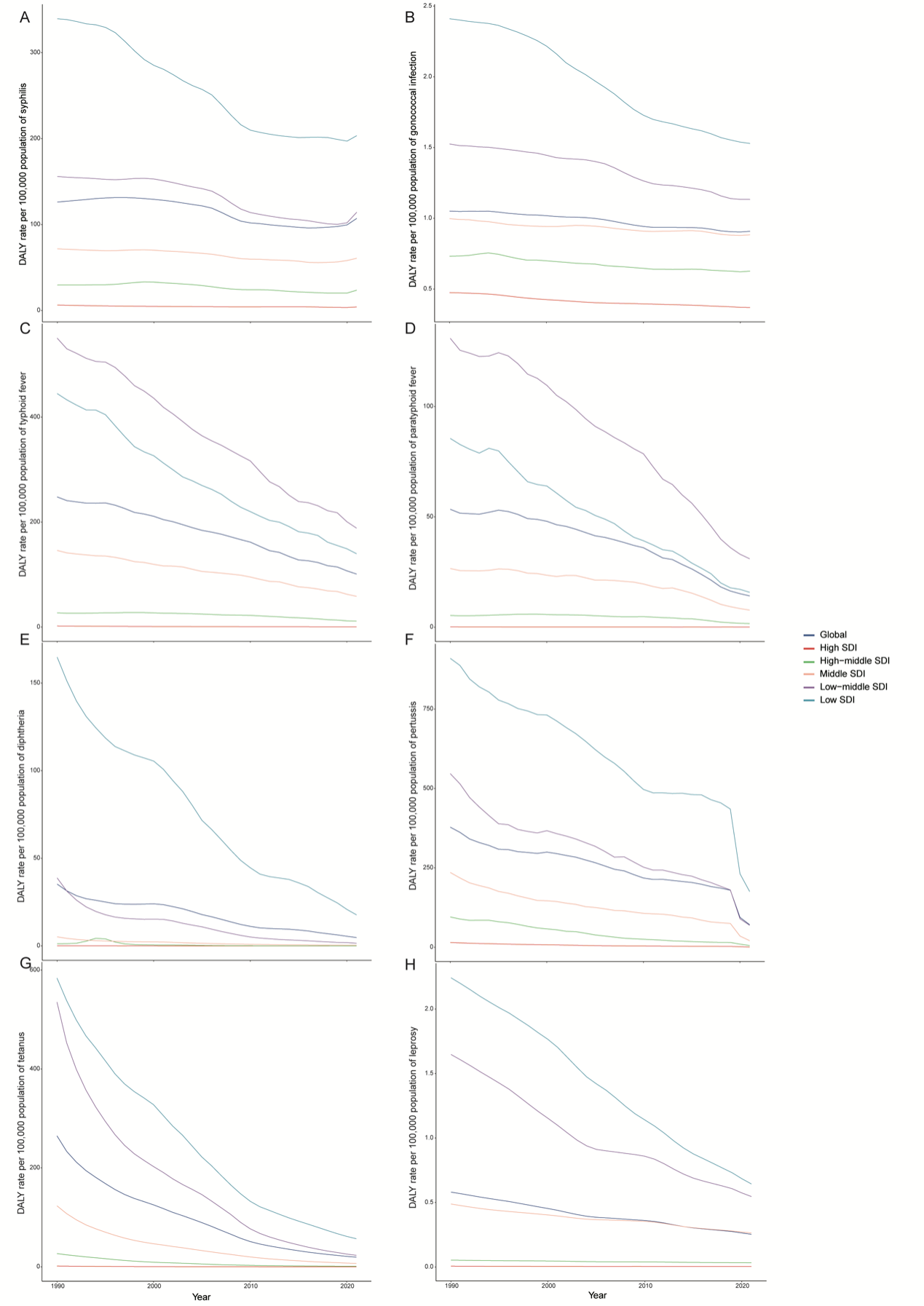
**

Fig. S3 Age-standardized DALY rates per 100,000 population of Syphilis (A), Gonococcal infection (B), Typhoid fever (C), Paratyphoid fever (D), Diphtheria (E), Pertussis (F), Tetanus (G) and Leprosy (H) from 1990 to 2021 by five SDI regions. (Abbreviations: DALY: disability-adjusted life year, SDI: Socio-demographic Index)

**
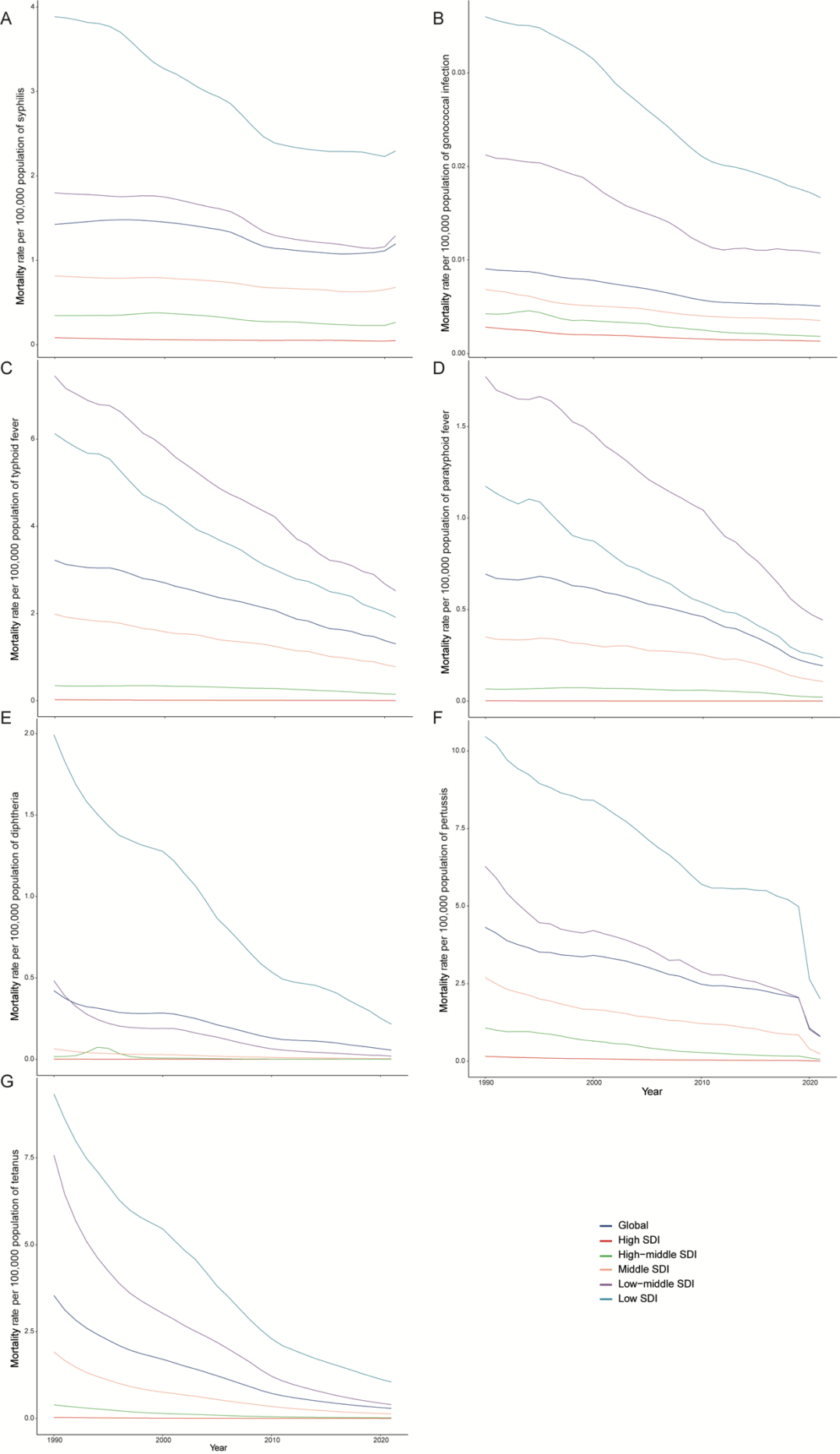
**

Fig. S4 Age-standardized mortality rates per 100,000 population of Syphilis (A), Gonococcal infection (B), Typhoid fever (C), Paratyphoid fever (D), Diphtheria (E), Pertussis (F), and Tetanus (G) from 1990 to 2021 by five SDI regions. (Abbreviation: SDI: Socio-demographic Index)

**
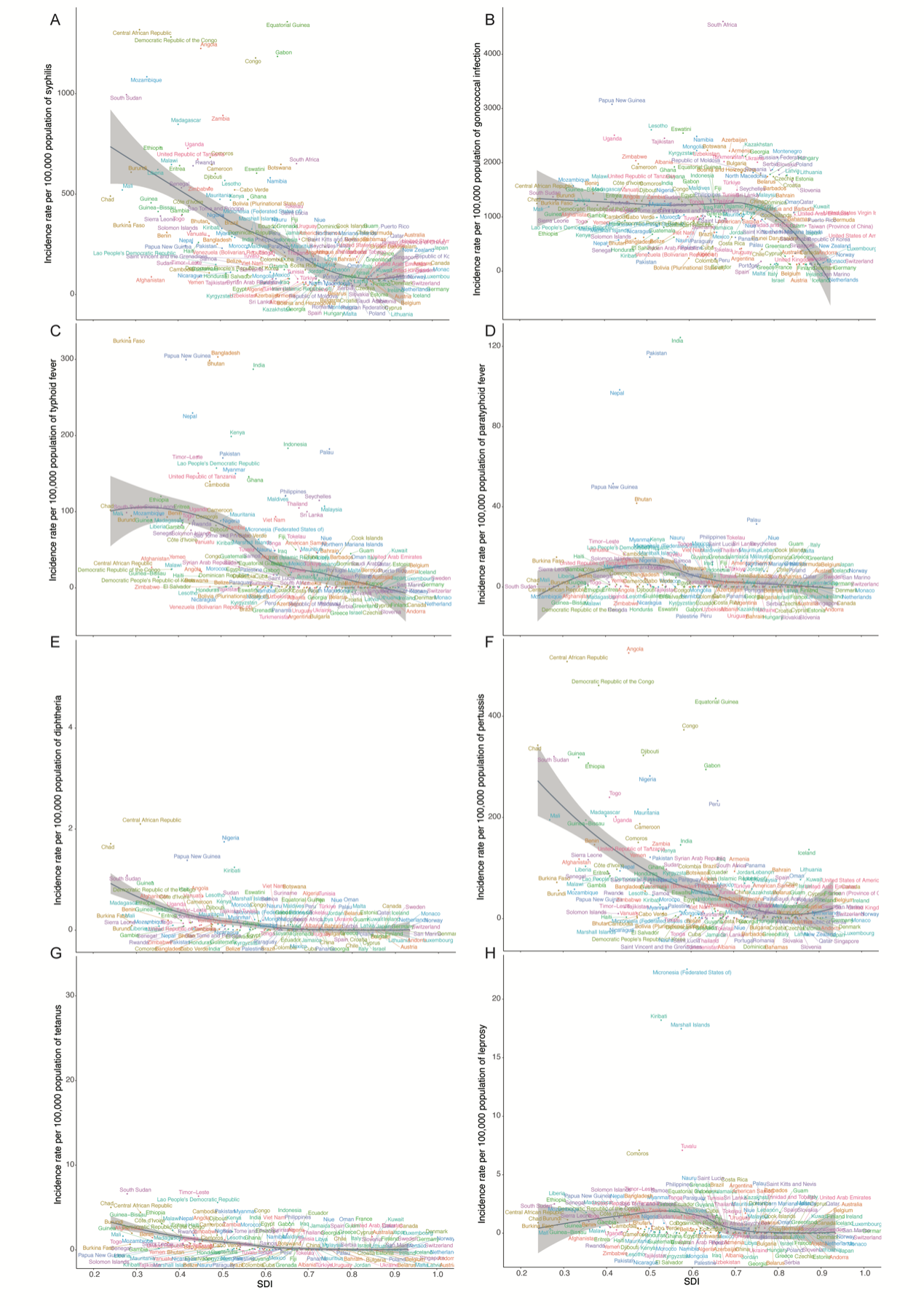
**

Fig. S5 Relationship between SDI and age-standardized incidence rates by country in 2021. Syphilis (A), Gonococcal infection (B), Typhoid fever (C), Paratyphoid fever (D), Diphtheria (E), Pertussis (F), Tetanus (G), Leprosy (H). (Abbreviation: SDI: Socio-demographic Index)


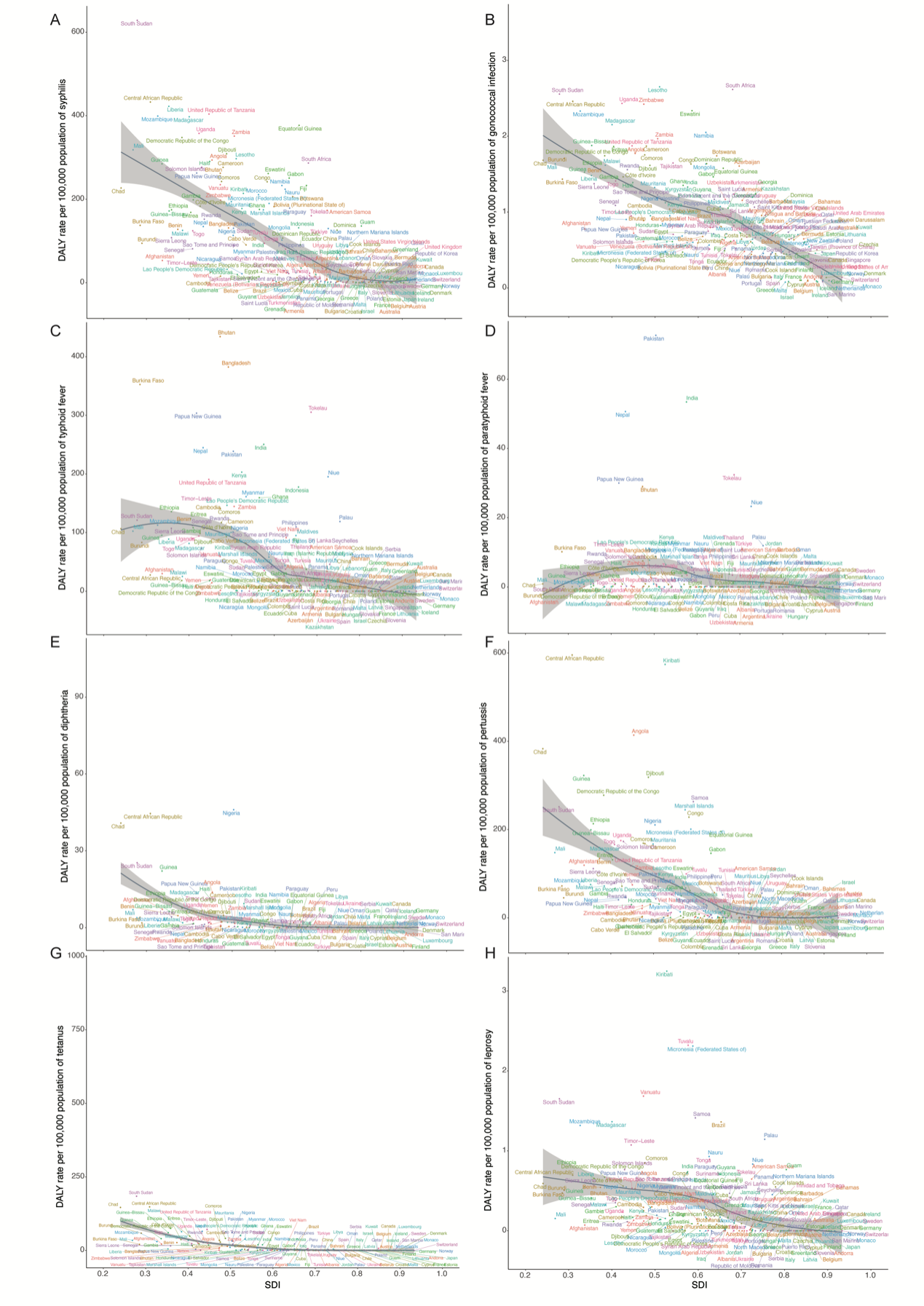


Fig. S6 Relationship between SDI and age-standardized DALY rates by country in 2021. Syphilis (A), Gonococcal infection (B), Typhoid fever (C), Paratyphoid fever (D), Diphtheria (E), Pertussis (F), Tetanus (G), Leprosy (H). (Abbreviations: DALY: disability-adjusted life year, SDI: Socio-demographic Index)

**
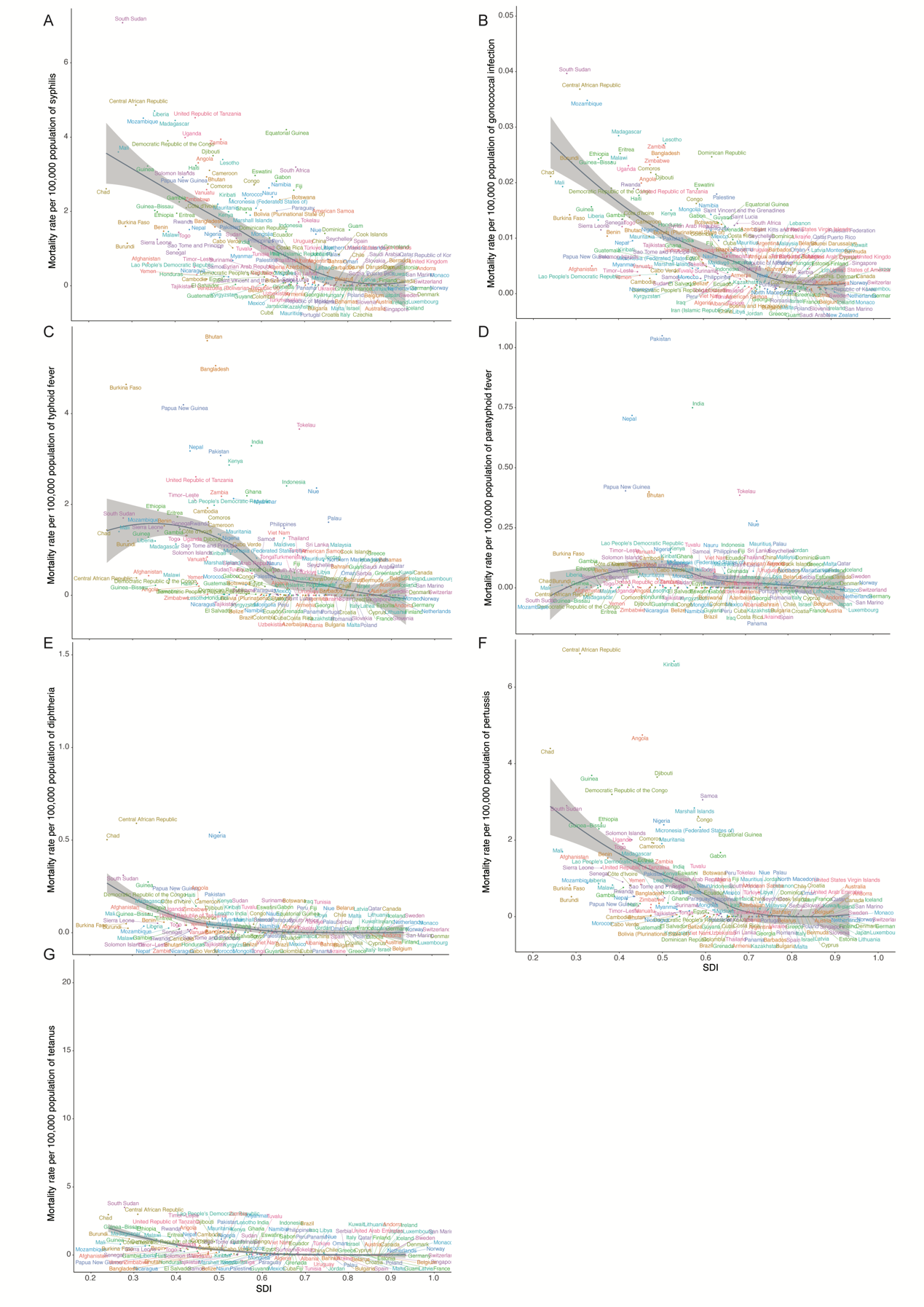
**

Fig. S7 Relationship between SDI and age-standardized mortality rates by country in 2021. Syphilis (A), Gonococcal infection (B), Typhoid fever (C), Paratyphoid fever (D), Diphtheria (E), Pertussis (F), Tetanus (G). (Abbreviation: SDI: Socio-demographic Index)

**
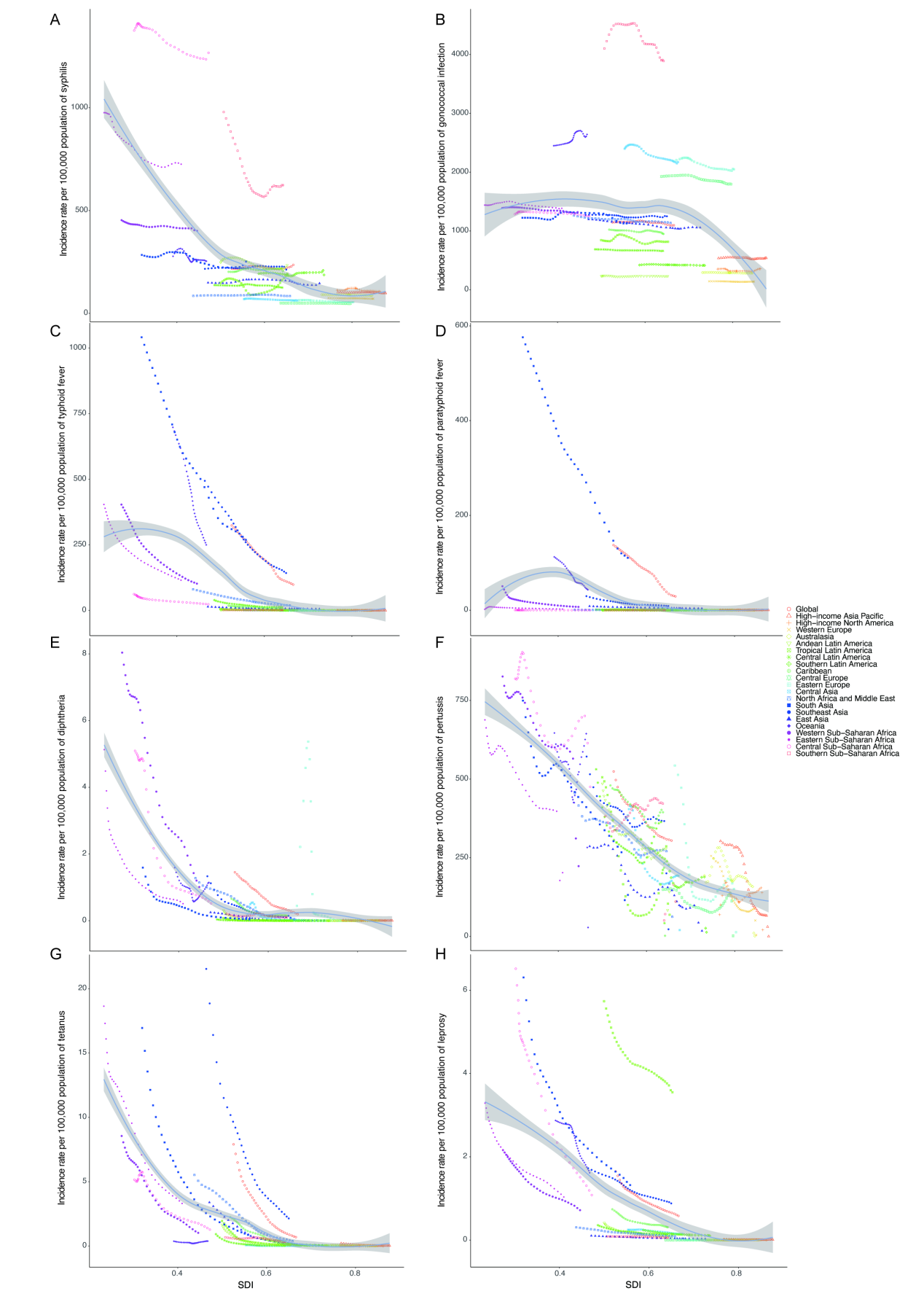
**

Fig. S8 Age-standardized incidence rates per 100,000 population of Syphilis (A), Gonococcal infection (B), Typhoid fever (C), Paratyphoid fever (D), Diphtheria (E), Pertussis (F), Tetanus (G) and Leprosy (H) for the 21 GBD regions by SDI, 1990-2021. (Abbreviations: GBD: Global Burden of Disease, SDI: Socio-demographic Index)


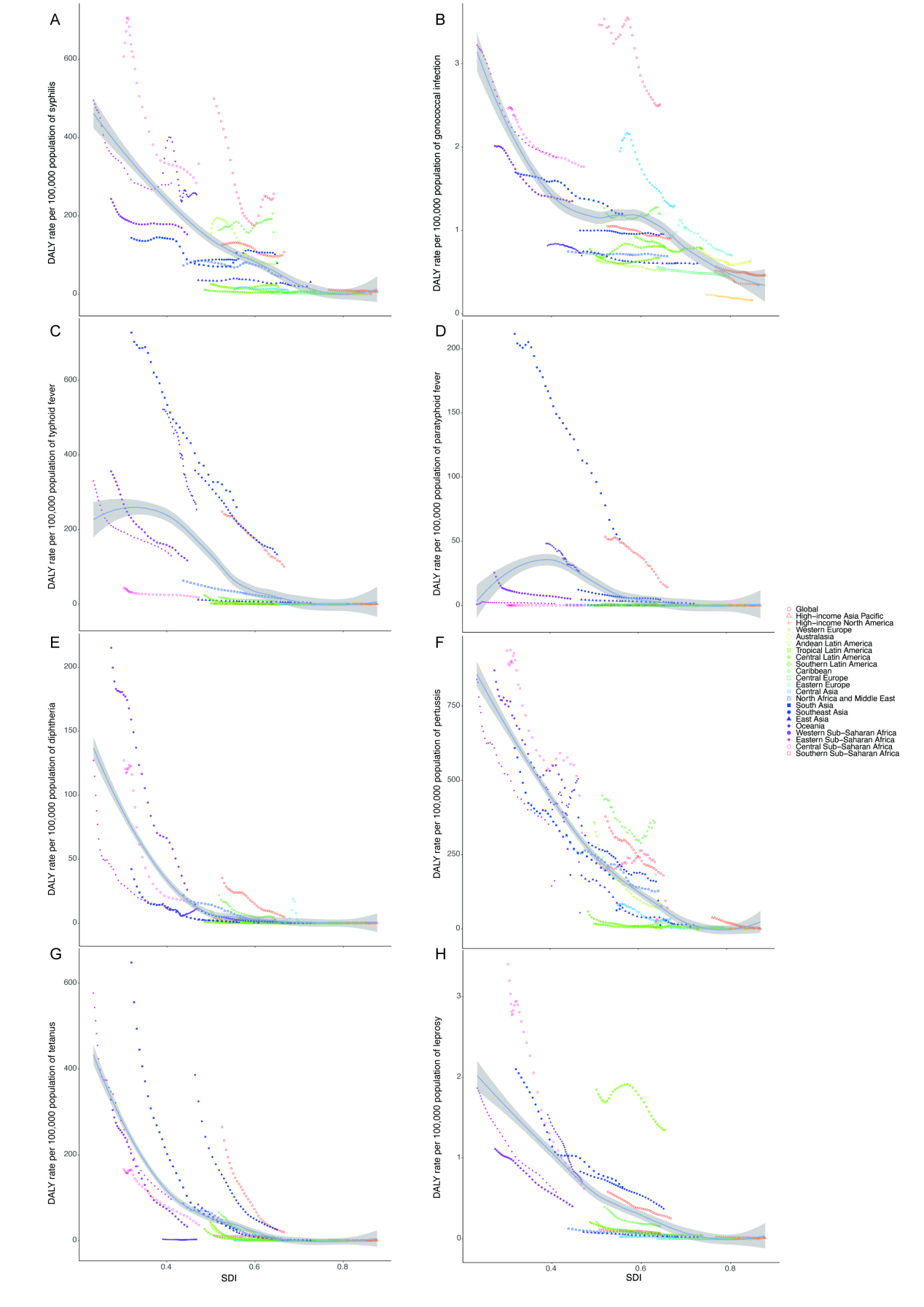
Fig. S9 Age-standardized DALY rates per 100,000 population of Syphilis (A), Gonococcal infection (B), Typhoid fever (C), Paratyphoid fever (D), Diphtheria (E), Pertussis (F), Tetanus (G) and Leprosy (H) for the 21 GBD regions by SDI, 1990-2021. (Abbreviations: DALY: disability-adjusted life year, GBD: Global Burden of Disease, SDI: Socio-demographic Index)

Fig. S10 Age-standardized mortality rates per 100,000 population of Syphilis (A), Gonococcal infection (B), Typhoid fever (C), Paratyphoid fever (D), Diphtheria (E), Pertussis (F), and Tetanus (G) for the 21 GBD regions by SDI, 1990-2021. (Abbreviations: GBD: Global Burden of Disease, SDI: Socio-demographic Index)
